# Transcriptional Integration of Distinct Microbial and Nutritional Signals by the Small Intestinal Epithelium

**DOI:** 10.1016/j.jcmgh.2022.04.013

**Published:** 2022-05-06

**Authors:** Colin R. Lickwar, James M. Davison, Cecelia Kelly, Gilberto Padilla Mercado, Jia Wen, Briana R. Davis, Matthew C. Tillman, Ivana Semova, Sarah F. Andres, Goncalo Vale, Jeffrey G. McDonald, John F. Rawls

**Affiliations:** 1 Department of Molecular Genetics and Microbiology, Duke Microbiome Center, Duke University School of Medicine, Durham, North Carolina; 2 Department of Cell Biology and Physiology, University of North Carolina at Chapel Hill, Chapel Hill, North Carolina; 3 Center for Human Nutrition, Department of Molecular Genetics, University of Texas Southwestern Medical Center, Dallas, Texas

**Keywords:** Chromatin, Microbiome, Lipid Metabolism, Intestine, ANOVA, analysis of variance, bp, base pair, CV, colonized, DHS, DNase hypersensitivity site, FA, fatty acid, FAO, fatty acid oxidation, GF, germ-free, GFP, green fluorescent protein, GO, Gene Ontology, HFM, high-fat meal, HNF4A, hepatocyte nuclear factor 4 alpha, IEC, intestinal epithelial cell, ISC, intestinal stem cell, LRT, likelihood ratio test, PCA, principal components analysis, PPARA, peroxisome proliferator activated receptor alpha, RNA-seq, RNA sequencing, ROI, region of interest, TF, transcription factor

## Abstract

**Background & Aims:**

The intestine constantly interprets and adapts to complex combinations of dietary and microbial stimuli. However, the transcriptional strategies by which the intestinal epithelium integrates these coincident sources of information remain unresolved. We recently found that microbiota colonization suppresses epithelial activity of hepatocyte nuclear factor 4 nuclear receptor transcription factors, but their integrative regulation was unknown.

**Methods:**

We compared adult mice reared germ-free or conventionalized with a microbiota either fed normally or after a single high-fat meal. Preparations of unsorted jejunal intestinal epithelial cells were queried using lipidomics and genome-wide assays for RNA sequencing and ChIP sequencing for the activating histone mark H3K27ac and hepatocyte nuclear factor 4 alpha.

**Results:**

Analysis of lipid classes, genes, and regulatory regions identified distinct nutritional and microbial responses but also simultaneous influence of both stimuli. H3K27ac sites preferentially increased by high-fat meal in the presence of microbes neighbor lipid anabolism and proliferation genes, were previously identified intestinal stem cell regulatory regions, and were not hepatocyte nuclear factor 4 alpha targets. In contrast, H3K27ac sites preferentially increased by high-fat meal in the absence of microbes neighbor targets of the energy homeostasis regulator peroxisome proliferator activated receptor alpha, neighbored fatty acid oxidation genes, were previously identified enterocyte regulatory regions, and were hepatocyte factor 4 alpha bound.

**Conclusions:**

Hepatocyte factor 4 alpha supports a differentiated enterocyte and fatty acid oxidation program in germ-free mice, and that suppression of hepatocyte factor 4 alpha by the combination of microbes and high-fat meal may result in preferential activation of intestinal epithelial cell proliferation programs. This identifies potential transcriptional mechanisms for intestinal adaptation to multiple signals and how microbiota may modulate intestinal lipid absorption, epithelial cell renewal, and systemic energy balance.

## Graphical abstract

**Figure:**
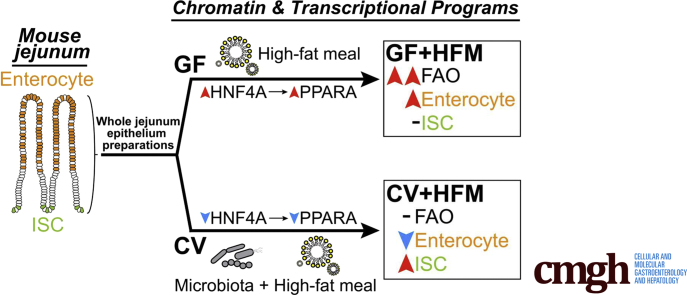

**Summary**

We identify genes and regulatory regions in mouse jejunum that conditionally respond to microbiota or high-fat meal. We find that these 2 stimuli interactively suppress a differentiated enterocyte program associated with fatty acid oxidation and mediated by HNF4A and PPARA.

The intestine simultaneously serves as the primary site for dietary nutrient absorption and as a habitat for resident microbiota. Microbial and nutritional signals are diverse, dynamic, and frequently simultaneous within the gut.^1^ Host diet can also dramatically influence microbial communities and their metabolites,^2^^,^^3^ and microbes can conversely modify nutritional signals.^4^ This allows microbes to influence existing host signaling pathways to modulate disease pathogenesis or create beneficial symbioses with the host.^4^, ^5^, ^6^, ^7^

Understanding how the intestine perceives and responds to the major stimuli of nutritional and microbial signals remains a fundamental challenge. Frequently, studies interrogate the effect of nutrients or microbes separately without exploring integrative host responses. For example, elevated levels of dietary fat have been shown to exert a dominant effect on energy intake and adiposity in mice^8^ and are implicated in the global prevalence of human metabolic disorders.^9^^,^^10^ However, there is ample evidence that high-fat diet and microbiota interactively influence host physiology. For example, germ-free (GF) mice are resistant to obesity from a high-fat diet.^2^^,^^11^, ^12^, ^13^

Whereas chronic high-fat diet feeding leads to adaptive physiological responses that can make it difficult to distinguish primary impacts of microbiota on host response,^14^^,^^15^ those impacts can be more easily discerned in the postprandial response to a single high-fat meal (HFM). Complementary studies in zebrafish and mice given a single HFM challenge have established that microbiota colonization promotes dietary fat absorption in intestinal epithelial cells (IECs) and distribution to the rest of the body.^2^^,^^16^, ^17^, ^18^ In both mice and zebrafish, different microbes appear to have heterogenous effects on lipid metabolism in IECs, implicating multiple pathways in the integration of these environments.^2^^,^^17^, ^18^, ^19^, ^20^ In support, in vitro exposure of mouse enteroids or an IEC line to several bacterial strains and their products has distinct effects on fatty acid (FA) metabolism and expression of associated genes.^2^^,^^19^^,^^20^ However, the mechanisms and pathways underlying these phenotypes in vivo remain unknown. Also, how genome-wide transcriptional responses in IECs integrate multiple signals simultaneously from a complex microbial community and diet remains unmapped.

Postprandial uptake of dietary lipids takes place primarily in the jejunum region of the small intestine. Genome-wide analyses of the intestinal response to microbes consistently show a reduction in expression of lipid metabolism genes in small intestinal epithelial cells and have implicated circadian rhythm, numerous transcription factors (TFs), and other regulators in the response including *Nfil3*, *Hdac3*, and lipid-liganded nuclear receptor TFs including *Ppara*, *Hnf4a*, and *Hnf4g*.^21^, ^22^, ^23^, ^24^, ^25^, ^26^, ^27^ Peroxisome proliferator activated receptor alpha (PPARA) functions as a major regulator of FA oxidation (FAO) genes and energy homeostasis partly through activating lipid metabolism genes when in the presence of lipids.^28^ Hepatocyte nuclear factor 4 alpha (HNF4A) is also a major regulator of lipid metabolism genes in the intestine and liver.^22^^,^^29^, ^30^, ^31^, ^32^, ^33^ In zebrafish, the majority of microbially suppressed genes, including many lipid metabolism genes, also lose expression in *hnf4a* mutants.^22^ In mouse, colonization results in a reduction of intestinal HNF4A occupancy at most sites across the genome.^22^ Collectively, this suggests HNF4A binding activity and function in IECs are relatively high in a GF context and may regulate the response to microbial and nutritional signals. However, the underlying reason for the overlap between microbial and *hnf4a*-regulated genes and how microbes alter HNF4A occupancy, host metabolism, and acquisition of nutrients remain unknown.

Here we applied multiple functional genomic assays to evaluate the interaction between HFM and microbiota colonization in mouse small intestinal IECs. Entry of a HFM into the small intestine initiates a dynamic postprandial process beginning with emulsification and digestion within the lumen, leading to uptake of lipids into IECs where they are temporarily stored in cytosolic lipid droplets, oxidized as a fuel source, or exported in chylomicron lipoproteins that are distributed via the circulatory system. We focused here on a single, early postprandial time point after gavage of a complex HFM consisting of chicken egg yolk emulsion, chosen to capture transcriptional responses during the initial perception and response to a complex HFM before cell-division and cell-type changes are substantial. Collectively, our results identify the chromatin-based and transcriptional interplay between microbial and nutritional responses in the jejunal epithelium and suggest microbes may promote lipid accumulation, weight gain, and proliferation by suppressing HNF4A, PPARA, and FAO in the intestine after a HFM.

## Results

### Characterizing Intestinal Adaptation to Microbes and HFM

**Figure 1:**
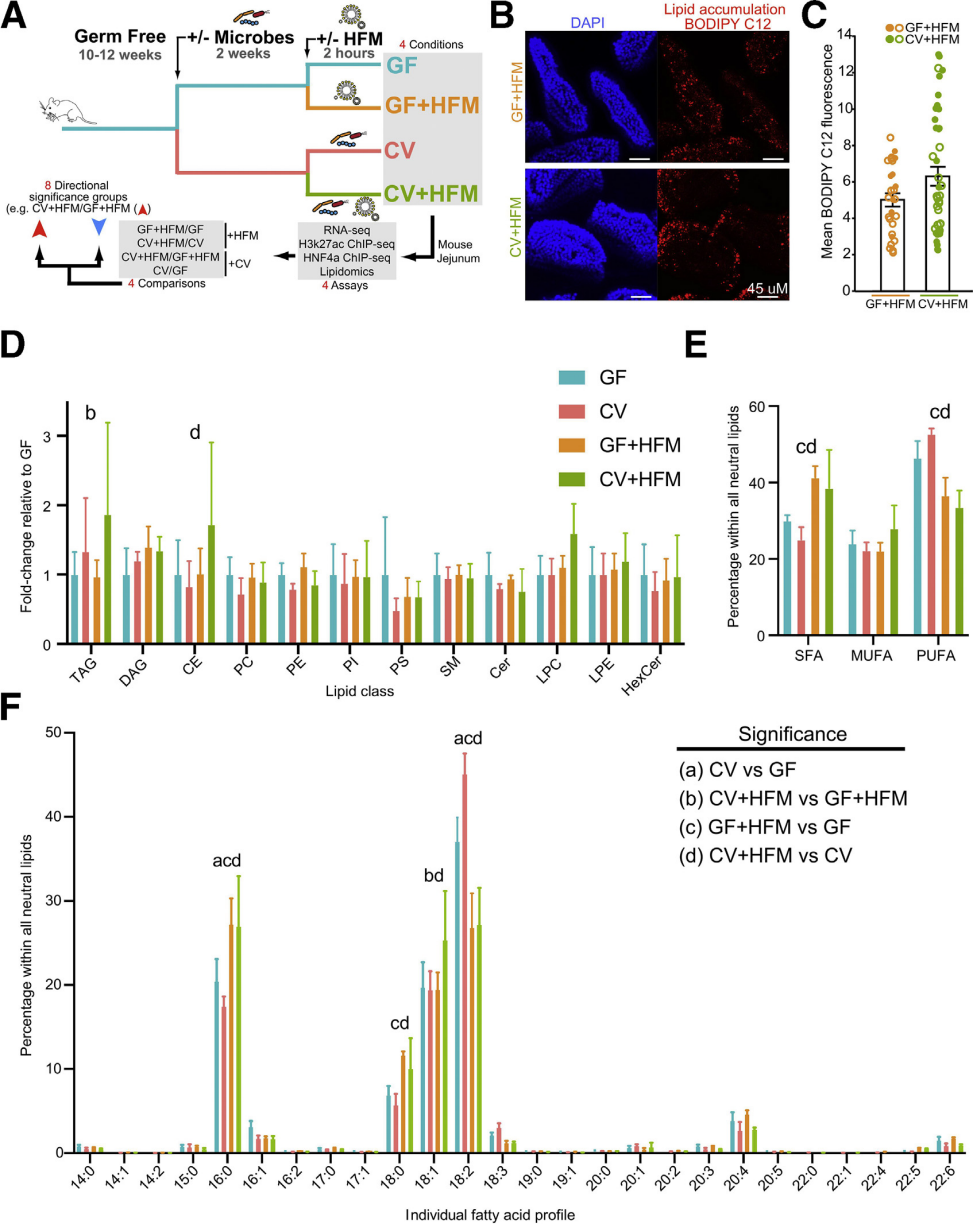
**Impact of HFM and microbes on the mouse intestine.** (*A*) Experimental schematic highlighting microbial and nutritional conditions, genomic assays, and analysis. (*B*) Confocal *en face* images of jejunal villi 2 hours after gavage with egg yolk labeled with BODIPY C12 (*red*) with nuclei labeled with DAPI (*blue*). (*C*) Quantification of mean BODIPY C12 fluorescence per villus. Data points represent individual villi (27 GF+HFM villi, 42 CV+HFM villi), with *open and closed circles* representing villi from 2 biological replicate mice per condition. Averages and standard deviations of villi measurements are shown. Student *t* test showed significant differences when comparing across villi (*P* = .023) but not mice (*P* = .304). (*D*) Relative abundance of major lipid classes in each sample type including neutral lipids (triacylglyceride [TAG]), diacylglyceride [DAG], cholesteryl ester [CE]), phospholipids (phosphatidylcholine [PC], phosphatidylethanolamine [PE], phosphatidylinositol [PI], phosphatidylserine [PS]), and polar lipids (sphingomyelin [SM], ceramide [Cer], lysophosphatidylcholine [LPC], lysophosphatidylethanolamine [LPE], hexosylceramides [HexCer]). All measurements were normalized to internal standards and are shown as fold change relative to GF. Two-way ANOVA revealed there was not a statistically significant interaction between treatment and lipid class (F_33,144_ = 0.8728, *P* = .6672). Simple main effects analysis showed that treatment did have a statistically significant effect on lipid class abundance (*P* = .0367). (*E*) Percentage of FAs detected across all neutral lipid classes (TAG, DAG, and CE) that are saturated FAs (SFA), monounsaturated FAs (MUFA), and polyunsaturated FAs (PUFA). Two-way ANOVA revealed there was a statistically significant interaction between treatment and FA saturation group (F_6,36_ = 13.93, *P* < .0001). Simple main effects analysis showed that treatment did not have a statistically significant effect on lipid class abundance (*P* > .9999). (*F*) Percentage of FAs detected across all neutral lipid classes (TAG, DAG, and CE) with the corresponding chain length and saturation. Two-way ANOVA revealed there was a statistically significant interaction between treatment and FA type (F_75,312_ = 10.98, *P* < .0001). Simple main effects analysis showed that treatment did not have a statistically significant effect on FA abundance (*P* > .9999). For the data shown in (D-F*)*, significant differences (*P* < .05) by post hoc Tukey multiple comparisons tests are noted for (a) GF vs CV, (b) GF+HFM vs CV+HFM, (c) GF vs GF+HFM, and (d) CV vs CV+HFM. Data are shown as average and standard deviation of 4 mice per condition. See also Supplementary Table 1.

By manipulating the presence of microbiota and HFM, we queried 4 conditions in the adult mouse jejunum: GF, GF+HFM, ex-GF colonized with a conventional microbiota for 2 weeks (colonized, CV), and CV+HFM (Figure 1*A*). Previous studies suggested that 2 hours after HFM gavage was sufficient to initiate lipid droplet accumulation and chylomicron export in IECs, but too early in the postprandial process to detect major differences in intestinal lipid transport between GF and CV mice.^2^^,^^12^^,^^34^ In accord, we found that gavaging mice with HFM consisting of a chicken egg yolk emulsion labeled with BODIPY-conjugated C12 FA led to BODIPY accumulation in jejunal IECs after 2 hours (Figure 1*B*). Wholemount confocal microscopy of villi from mice in each +HFM condition identified an increase in epithelial BODIPY signal in CV+HFM relative to GF+HFM, which was significant on the basis of a signal per villi measurement, but not when comparing across mice (Figure 1*C*). This is consistent with previous reports that microbial colonization promotes lipid accumulation in IECs^2^^,^^18^^,^^19^ and confirms that lipid has reached jejunal IECs by this time point. We next performed direct infusion MS/MS^ALL^ lipidomic analysis^35^ on jejunal IEC preparations (see Methods) to assess potential differences in lipid content under these 4 conditions. Comparing the relative abundance of major lipid classes in the 4 conditions revealed a small significant increase of triacylglycerol in CV+HFM compared with GF+HFM, consistent with increased lipid absorption in CV (Figure 1*D*, Supplementary Table 1). Analysis of FA saturation within the entire neutral lipid pool (i.e., triacylglycerols, diacylglycerols, and cholesteryl esters) revealed relative increases in the abundance of saturated FA in both HFM conditions (38% increase relative to GF [*P* = .0051] and 53% increase relative to CV [*P* = .0009]) and relative decreases in polyunsaturated FA (20% decrease relative to GF [*P* = .0224] and 36% decrease relative to CV [*P* < .0001]; Figure 1*E*). Analysis of individual FA species within the neutral lipid pool suggested these differences were driven largely by relative increases in the abundance of FA 16:0 and 18:0 in both HFM conditions and relative decreases in FA 18:2 (Figure 1*F*), presumably reflecting the influx of saturated FA that predominates in chicken egg yolk.^36^ Analysis of other FA species and broader lipid classes suggested other potential impacts of microbiota and HFM feeding (Figure 1*F*, Supplementary Table 1). Together these results establish that 2 hours of HFM feeding is sufficient to initially incorporate dietary lipids into IECs, with only minor differences in lipid content between GF and CV states at this early postprandial stage.

### Transcriptional Changes Integrate Microbial and Nutritional Responses in the Intestine

**Figure 2:**
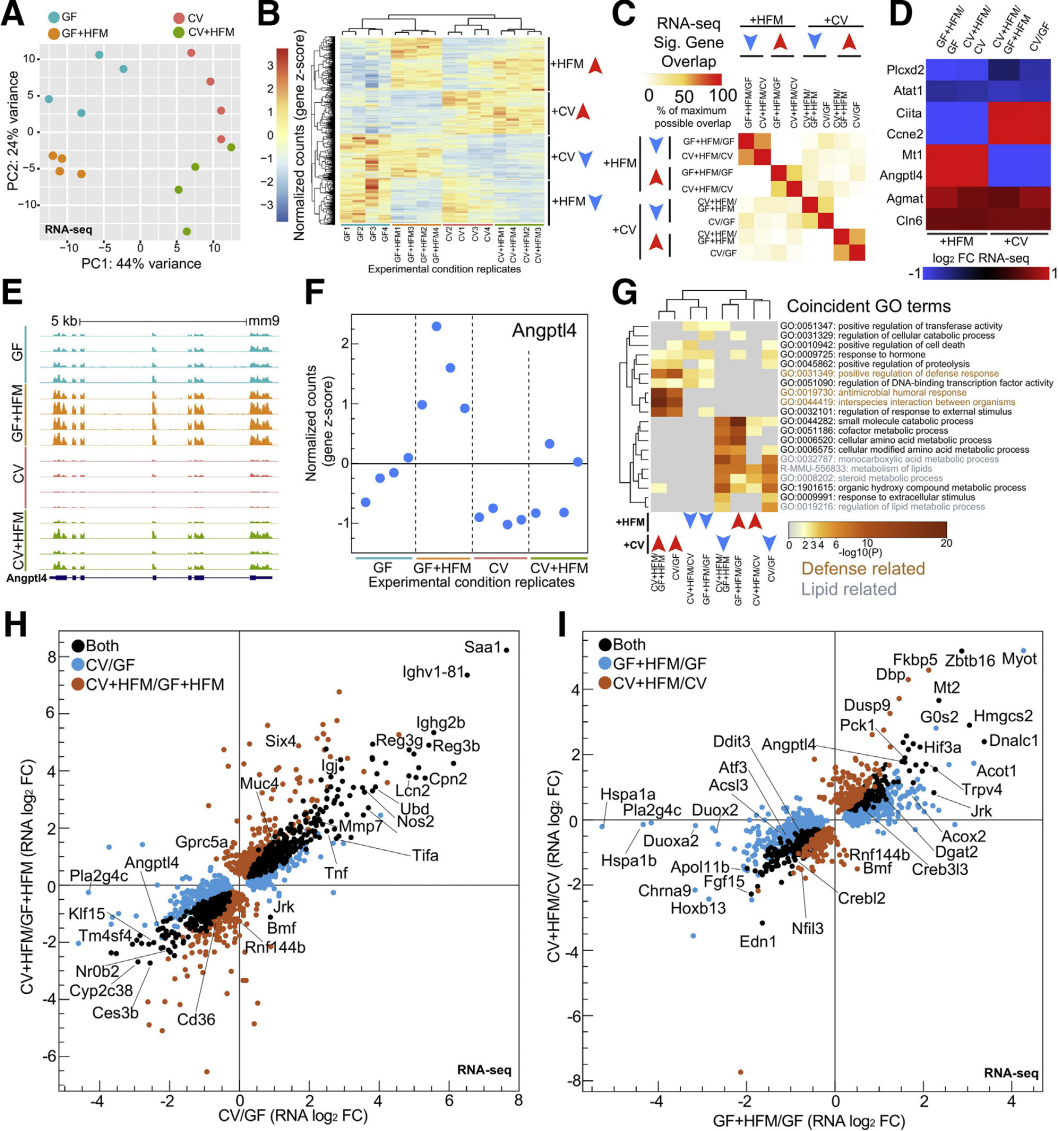
**Impact of HFM and microbes on gene transcription in the mammalian intestine.** (*A*) DESeq2 PCA of RNA-seq normalized counts in each replicate for the 4 conditions, with 4 mice per condition. Adonis permutational multivariate ANOVA of RNA-seq distance matrix; microbes: *P* = .002, R2 = 0.240; meal: *P* = .005, R2 = 0.169. (*B*) Heatmap of row z-scored normalized counts for genes significantly differential in at least one comparison by RNA-seq (*P* adjusted < .05). Examples of blocks of commonly behaving genes are marked for +HFM and +CV directional groups. (*C*) Pairwise comparison of maximum overlap of coincident significant RNA-seq genes (*P* adjusted <.05) for 8 directional significance groups shows generally coincident directionality and genes for +CV and +HFM comparisons. (*D*) Heatmap of example genes significantly different in both a +CV and +HFM comparison (*P* adjusted <.05). (*E*) UCSC screenshot for RNA-seq replicate levels at the mouse *Angptl4* locus. (*F*) RNA-seq z-scored normalized counts of *Angptl4*, which are significant in both +CV and +HFM comparisons, show amplified relative expression in the GF+HFM condition. (*G*) Clustered heatmap of significance values for shared GO terms in at least 2 of 8 directional RNA-seq significance groups. (*H*) Scatterplot of significantly different gene log_2_ fold change for CV/GF and CV+HFM/GF+HFM RNA-seq (*P* adjusted <.05). (*I*) Same as (*H*) for GF+HFM/GF and CV+HFM/CV (*P* adjusted <.05).

RNA sequencing (RNA-seq) of unsorted jejunal IEC preparations (see Methods) showed the 4 conditions with each experimental replicate clustering based on treatment (Figure 2*A*). We identified hundreds of genes with significant transcriptional differences for each of the separate 4 comparisons (+CV: CV/GF and CV+HFM/GF+HFM and +HFM: GF+HFM/GF and CV+HFM/CV) (Figure 1*A*, Supplementary Table 2) and identified blocks of genes that were primarily impacted by either microbial or nutritional status (Figure 2*B*). Overlap of differential genes within both +HFM comparisons or both +CV comparisons identified substantial agreement (Figure 2*C*). However, we noted that numerous genes were also significantly different in response to both +CV and +HFM conditions, suggesting +CV and +HFM stimuli were both integrated at a subset of genes (Figure 2*D*, Supplementary Table 2). These genes include angiopoietin-like 4 (*Angptl4*), a secreted lipoprotein lipase inhibitor involved in partitioning triglyceride availability and lipid accumulation known to be suppressed by microbiota^11^^,^^12^^,^^22^^,^^37^ and activated by fasting and high-fat diet.^38^^,^^39^ *Angptl4* showed significant transcriptional differences in all 4 comparisons; it was elevated in GF relative to CV and further up-regulated by HFM in both microbial conditions (Figure 2*D–F*). Therefore, the nutritional and microbial signals that regulate gene transcription, while separable, can be additive or subtractive in their contributions to regulate transcription within the intestine**.**

We next used Gene Ontology (GO) terms to functionally categorize each of the comparisons broken down into genes that were up- or down-regulated to create 8 total differential significance groups (Figure 2*G*, Supplementary Table 3). Surprisingly, +CV–down-regulated groups’ GO terms were clustered and similar to +HFM–up-regulated groups for terms such as metabolism of lipids and steroid metabolic process (GO term genes include *Cyp27a1*, *Acaa2*, *Acox2*, *Acsl1*, *Acaa1a*). Conversely, +HFM-down and +CV-up groups shared positive regulation of defense response (*Nfkbiz*, *Duoxa2*, *Tnfrsf11a*), suggesting for certain processes, there may be genes regulated by both nutritional and microbial inputs (Figure 2*G*).

To determine whether any of the changes in gene expression varied on the basis of colonization and nutritional status, we characterized the +CV (CV/GF and CV+HFM/GF+HFM, Figure 2*H*) and +HFM (GF+HFM/GF and CV+HFM/CV, Figure 2*I*) responses separately. We observed that for most genes the magnitude and directionality of changes are consistent and positively correlated in the 2 +CV comparisons (Figure 2*H*). Previously characterized microbially responsive defense genes such as *Saa1*, *Reg3g*, and *Tnf* were up-regulated in both +CV comparisons (Figure 2*H*, Supplementary Table 2).^22^ Numerous metabolism genes were commonly down-regulated by microbes including previously characterized microbially responsive genes *Angptl4*, *Nr0b2*, *Klf15*, and *Ces3b* (Figure 2*H*).

The genes that respond to HFM exposure with and without microbes followed a similar positive correlation of RNA log_2_ fold changes (Figure 2*I*). *Hmgcs2, Angptl4*, *Creb3l3*, *Acot1*, and *Dgat2*, all involved in lipid metabolism, were commonly up-regulated in both +HFM comparisons (Figure 2*I*). GO term analysis in +HFM-up genes identified enrichment for metabolism of lipids, peroxisome, and PPAR signaling (Supplementary Table 3). Genes down-regulated by HFM included genes involved in the unfolded protein response (*Ddit3*, *Atf3*), TFs (*Nfil3*, *Crebl2*, *Klf6*, *Fosb*), signaling (*Fgf15, Fzd7*, *Jag1*, *Wnt5a*), and inflammatory components (*Nfkbiz*, *Nlr9b*, *Duox2*, *Duoxa2*).

### Influence of Microbial and HFM Signals at FAO Genes

**Figure 3:**
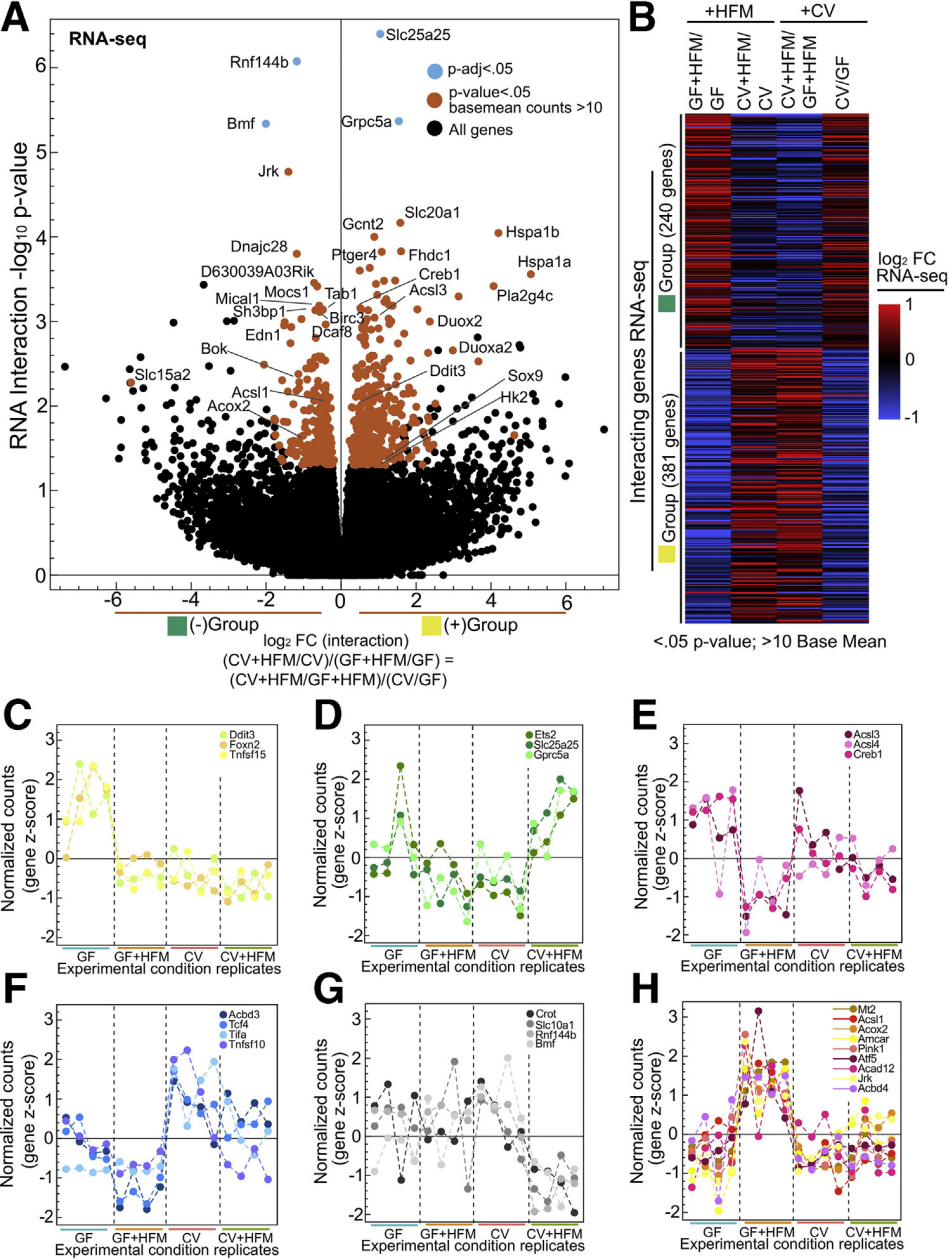
**Characterizing putative transcriptional interaction genes.** (*A*) Volcano plot showing interaction log_2_ fold change versus –log_10_*P* value for typical (*P* adjusted <.05; *blue*) and lenient (*P* value <.05, >10 base mean counts; *red*) cutoffs identifies genes with greatest potential for interaction. In effect, the interaction log_2_ fold change represents the log_2_ ratio of (CV+HFM/CV)/(GF+HFM/GF) or (CV+HFM/GF+HFM)/(CV/GF), which are equivalent because these comparisons contain the same 4 conditions. Because negative (–, *green*) and positive (+, *yellow*) interactions are representative of the directionality of the fold change but not necessarily the nature of the interactions, these groups are also colored to help illustrate that property. (*B*) Heatmap of log_2_ FC for each comparison for interaction genes broken into the green and yellow groups. (*C–H*) RNA-seq z-scored normalized counts for example interacting genes, with each panel showing a different expression pattern.

To test more specifically for interaction between microbes and HFM and identify resultant gene expression patterns, we performed likelihood ratio test (LRT) analysis on our RNA-seq dataset across all conditions (Figure 3*A*, Supplementary Table 2).^40^ Although only a limited number of genes (*Rnf144b*, *Slc25a25*, *Grpc5a*, *Bmf*) passed a *P* adjusted threshold of .05 for the LRT analysis, we proceeded to lower this cutoff to identify 621 genes with the most potential for interaction (Figure 3*A* and *B, see Methods*). Typically, putative interaction genes showed substantial increased or decreased relative expression in one condition (Figure 3*C–H*). In GF+HFM there was a reduction in *Creb1*, *Acsl4*, and the lipid droplet promoting *Acsl3* (Figure 3*E*). This was in contrast to the up-regulation of FAO genes *Acsl1*, *Acox2*, *Amcar*, and *Acad12* in GF+HFM, suggesting the FAO pathway is reduced by microbes and induced by HFM at the transcript level (Figure 3*H*).

### Microbial and Nutritional Stimuli Signal to Many of the Same Intestinal Regulatory Regions

**Figure 4:**
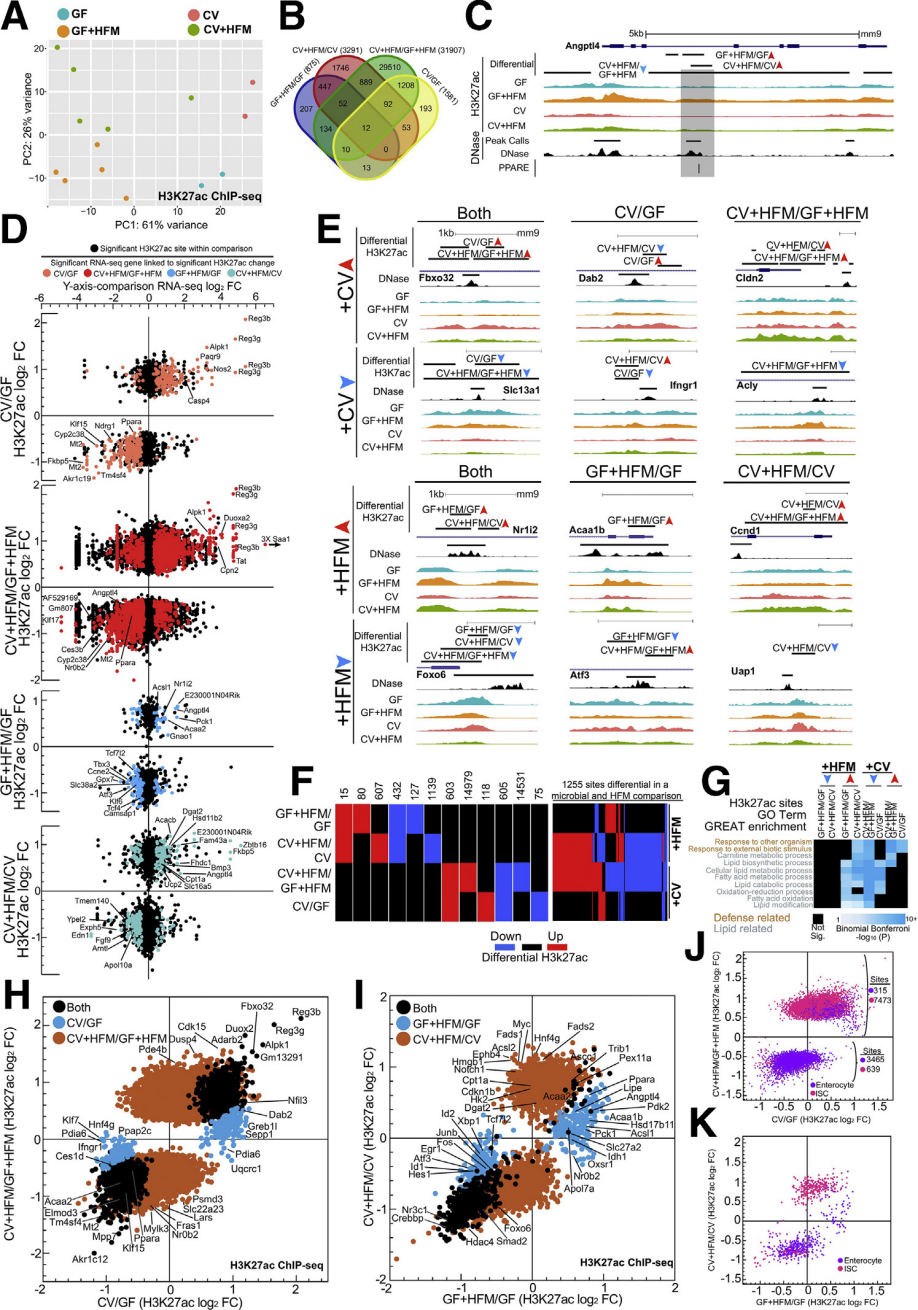
**Identification of nutritional and microbial regulatory regions in the mammalian intestine.** (*A*) DESeq2 PCA of H3K27ac ChIP-seq normalized counts for all replicates for each condition. Replicates represent individual mice: CV = 2, CV+HFM = 5, GF = 2, and GF+HFM = 5. Adonis permutational multivariate ANOVA of H3K27ac distance matrix; microbes: *P* = .002, R2 = 0.234; meal: *P* = .102, R2 = 0.111. (*B*) Venn diagram of overlap for significant H3K27ac sites for +CV and +HFM comparisons (*P* adjusted <.05). (*C*) Average H3K27ac ChIP-seq and DNase-seq signal for various conditions at the *Angptl4* locus. An accessible chromatin region coincident with a characterized PPAR binding site in intron 3 was microbially suppressed and +HFM induced.^43^ (*D*) Scatterplots of RNA-seq versus H3K27ac log_2_ fold change for all 4 comparisons using the single nearest gene neighboring the significantly differential H3K27ac site (*P* adjusted <.05). *Colored dots* represent associated genes significant by RNA-seq in that comparison (*P* adjusted <.05) and associated with differential H3K27ac sites. (*E*) Example loci showing significantly differential H3K27ac enrichment for various comparisons including across multiple comparisons. (*F*) Quantification of different patterns of differential H3K27ac DNase sites (*P* adjusted <.05). The number of sites is enumerated above the pattern for each group that is significantly differential in either a +CV or +HFM comparison (*left*). One thousand two hundred fifty-five regulatory regions are responsive in both a +CV and +HFM comparison (*right*). (*G*) Coincident GREAT GO terms enrichment for 8 H3K27ac directional significance groups.^44^ (*H*) Scatterplot of average H3K27ac sites with significantly different log_2_ fold change window for CV/GF and CV+HFM/GF+HFM (*P* adjusted <.05). (*I*) Same as (*H*) for GF+HFM/GF and CV+HFM/CV. (*J*) Scatterplot of significant H3K27ac sites for CV/GF and CV+HFM/GF+HFM log_2_ FC colored by overlap with enterocyte (*purple*) or ISC (*pink*) regulatory regions. (*K*) Same as (*J*) for GF+HFM/GF versus CV+HFM/CV.

H3K27ac modification of nucleosomes flanking accessible regulatory regions is associated with activation of transcription of neighboring genes. Analysis of these sites in multiple conditions allows for robust interpretation of the genes, TFs, and pathways involved in coordinated transcriptional regulation.^22^^,^^41^^,^^42^ We identified H3K27ac levels genome-wide in jejunal IECs in the 4 conditions. As we observed in our RNA-seq (Figure 2*A*), H3K27ac ChIP-seq replicates grouped on the basis of the conditions along the PC1 and PC2 axis by principal components analysis (PCA); however, groups were not significantly different on the basis of HFM at this level (Figure 4*A*). An increased number of replicates for CV+HFM and GF+HFM relative to CV and GF improved the ability to identify differential H3K27ac CV+HFM/GF+HFM sites but also led to more significant sites relative to other comparisons (Figure 4*B*, Supplementary Table 4). Similar to our RNA-seq, the *Angptl4* locus showed a region in intron 3 with both microbially reduced and HFM induced H3K27ac levels, with GF+HFM having the highest relative level of H3K27ac (Figure 4*C*). Diverse patterns of H3K27ac enrichment linked to RNA levels of neighboring genes were also found at promoter, intragenic, and intergenic regions (Figure 4*D* and *E*; two-sided Kolmogorov-Smirnov test for each comparison, *P* < .05)**.** Although many sites only significantly changed in either +CV or +HFM comparisons, 1255 regulatory regions integrate signals from both microbial and HFM conditions (Figure 4*F*). Similar to RNA-seq, we found shared enriched GO terms across +CV and +HFM comparisons. +HFM-up and +CV-down sites were nearest to genes related to lipid metabolism, and response to other organisms was found in +HFM-down and +CV-up groups (Figures 2*G* and 4*G*, Supplementary Table 3).

### Sites With Increased H3k27ac After HFM Behave Differently Depending on the Presence of Microbiota

Having established the utility of our H3K27ac data, we proceeded to compare significant log_2_ fold change levels for H3K27ac sites differential for the 2 +CV and 2 +HFM comparisons (Figure 4*H* and *I*). Like RNA-seq (Figure 2*H*), we observed a general positive correlation for relative H3K27ac log_2_ fold change levels in the 2 +CV comparisons (Figure 4*H*). +CV sites with increased H3K27ac enrichment were linked to known microbial transcriptionally responsive genes such as *Reg3g*, *Reg3B*, *Saa1*, and *Duox2.*^22^ The regulatory regions in both +CV-down H3K27ac comparisons neighbored lipid metabolism genes. These included FAO components such as *Acaa2*, *Ppara*, *Acsl5*, and *Slc27a4* and TFs *Klf15*, *Nr1h4*, *Zbtb16*, and *Id2* (Supplementary Table 3).^21^^,^^22^^,^^26^

However, unlike our RNA-seq analyses, we identified an unexpected separation only at sites that show increased H3K27ac enrichment after HFM, with surprisingly few sites having significance in both GF+HFM/GF-up and CV+HFM/CV-up comparisons (Figure 4*I*). The GF+HFM/GF-up only H3K27ac sites included lipid metabolism genes such as *Acsl1*, *Acaa1b*, *Apoa1*, *Lipe*, and *Crot* (Figure 4*I*, Supplementary Table 3). CV+HFM/CV-up only H3K27ac sites also included lipid metabolism related genes *Cpt1a*, *Cpt2*, *Ppard*, *Dgat2*, *Fads1*, *Fads2*, *Fasn*, and *Lipg*, many of which are associated with lipid anabolism^30^ (Figure 4*I*, Supplementary Table 3). Although not represented by a particular GO term, we also noted many CV+HFM/CV-up regulatory regions neighbor genes with known roles in intestinal proliferation and intestinal stem cell (ISC) identity including *Myc*, *Notch1*, *Ephb4*, *Ccnd1*, *Acsl2*, and *Sox9* (Figure 4*I*, Supplementary Table 3). To interrogate the potential impact of a change in chromatin-associated proliferation markers, we overlapped our regulatory regions with previously identified accessible regulatory sites from separately purified populations of ISCs and enterocytes.^45^ We found enterocyte-associated regulatory regions had reduced enrichment, and ISC-associated regulatory regions had increased enrichment after colonization (Figure 4*J*). Interestingly, we saw that the separation at sites with increased enrichment after HFM resulted in CV+HFM/CV up sites overlapping with ISC-associated regulatory regions and GF+HFM/GF up sites overlapping enterocyte-associated regulatory regions (Figure 4*K*). This suggests that a major impact of intestinal adaptation to nutritional and microbial stimuli is alteration of enterocyte and ISC regulatory regions and that different signals may be integrated at these sites.

**Figure 5:**
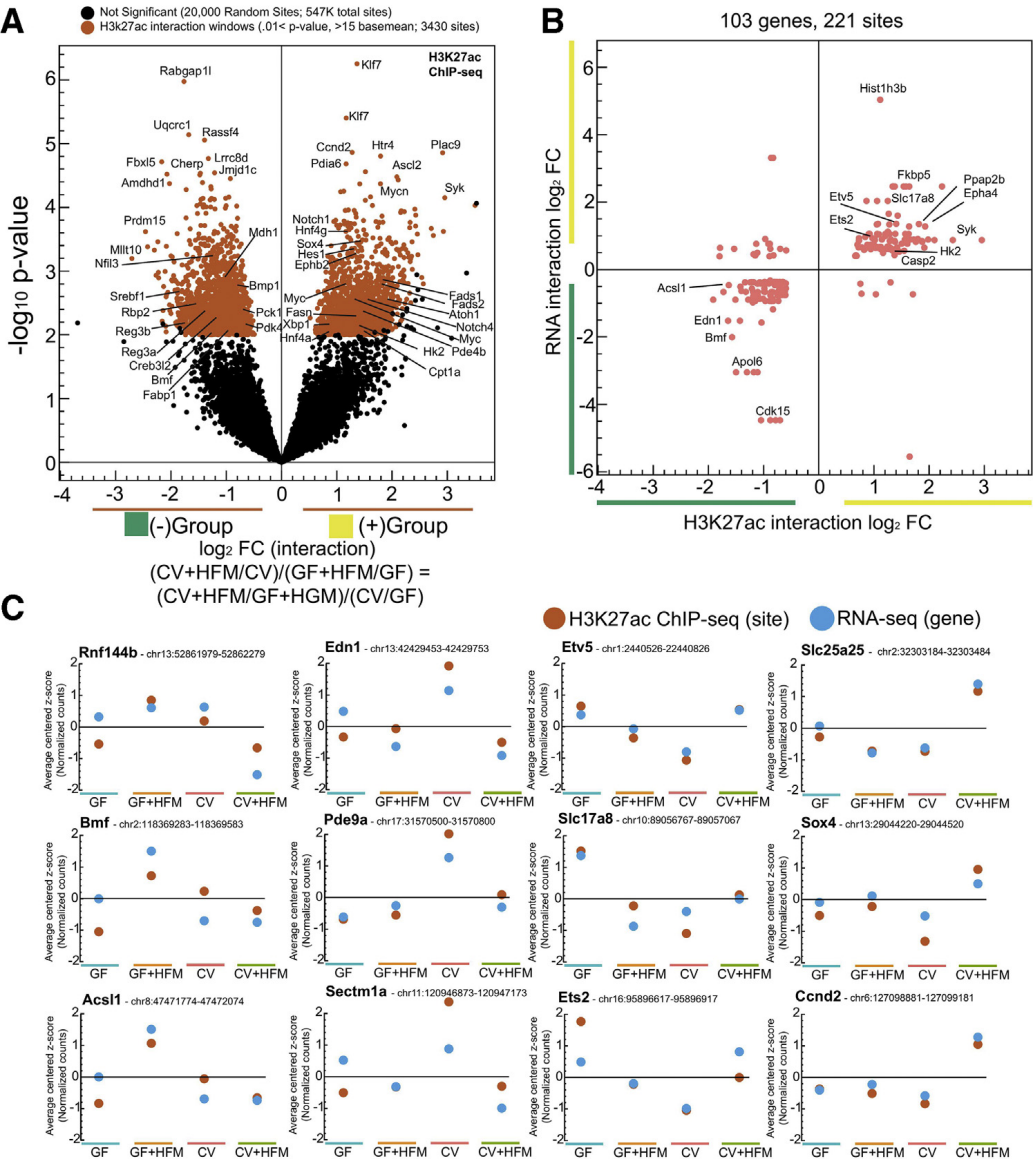
**Characterizing putative interaction regulatory regions.** (*A*) Volcano plot showing interaction log_2_ fold change versus –log_10_*P* value for lenient (*P* value <.01, >15 base mean; *red*) cutoffs identifies H3K27ac regulatory windows with greatest potential for interaction. Twenty thousand out of 547,000+ enriched H3K27ac windows that did not pass the lenient interaction cutoff were chosen at random to represent noninteracting sites. (*B*) Scatterplot comparing H3K27ac log_2_ interaction windows fold change linked to the RNA log_2_ interaction fold change for genes that also show interaction reveals a positive correlation suggesting many of these regions are causal in contributing to the transcription patterns of these genes across +CV and +HFM conditions. (*C*) Selected putative H3K27ac interaction windows from each cluster showing consistent patterns of H3K27ac enrichment and relative RNA levels across the 4 conditions.

Like RNA-seq, we also used LRT to characterize H3K27ac sites and found limited strong interactions. We therefore used a lenient statistical cutoff to identify 3430 H3K27ac sites with the greatest evidence of interaction (Figure 5*A*, Supplementary Table 5, see Methods). Comparison between H3K27ac putative interaction sites linked to genes that also had putative transcriptional interactions revealed 89.1% (197/221) were in agreement and commonly showed the same pattern across conditions (Figure 5*B* and *C*). This implies these functional processes of H3K27ac modification and gene transcription are linked, including in the regulation of divergent lipid metabolic processes and impacts on proliferation and ISCs, and are engaged differentially in response to HFM depending on microbiota colonization.

### Differential Integration of Both Microbial and Nutritional Signals at the Same PPARA/FAO and ISC Regulatory Regions

**Figure 6:**
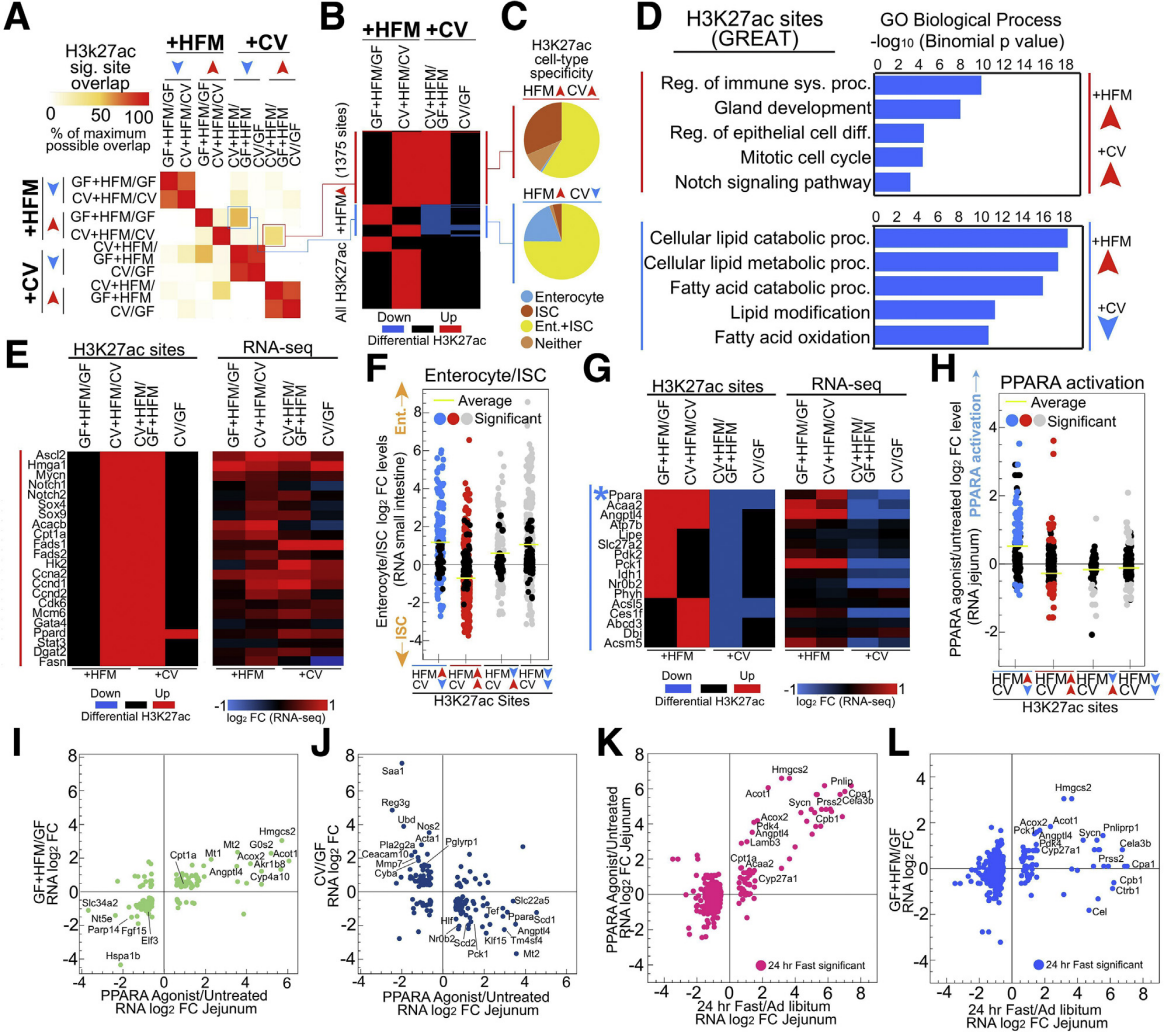
**Microbial and nutritional stimuli signal to many of the same intestinal regulatory regions.** (*A*) Pairwise comparison of maximum overlap of coincident significant H3K27ac sites (*P* adjusted <.05) for 8 directional significance groups identifies microbially responsive regulatory regions with CV+HFM/CV-up also being CV+HFM/GF+HFM-up (*red*) and GF+HFM/GF-up also being CV+HFM/GF+HFM-down (*blue*). (*B*) Heatmap of differential comparisons (*P* adjusted <.05) for all +HFM-up sites shows the proportion that is also microbially responsive. (*C*) Pie charts for red and blue +HFM-up groups that show the proportion that overlap with previously characterized enterocyte and ISC regulatory regions.^45^ (*D*) GREAT GO term enrichment for red and blue +HFM-up H3K27ac groups. (*E*) Heatmap of example red +HFM-up and +CV-up H3K27ac sites and their linked gene’s RNA-seq log_2_ fold change, including many loci associated with ISCs and proliferation. (*F*) Different combinations of differential H3K27ac sites that are both +HFM and +CV responsive show that only red sites that are +HFM-up and +CV-up are linked to genes that are preferentially expressed in ISCs relative to enterocytes. (*G*) Heatmap of example blue +HFM-up H3K27ac sites and their linked gene’s RNA-seq log_2_ fold change. *Blue asterisk* marks *Ppara* regulatory region that is characterized in Figure 7. (*H*) Different combinations of differential H3K27ac sites that are both +HFM and +CV responsive show that only blue sites that are +HFM-up and +CV-down are linked to genes that are activated by PPARA. (*I*) Scatterplot of genes that are significantly differential after PPARA activation and in GF+HFM/GF show a positive correlation. (*J*) Scatterplot of genes that are significantly differential after PPARA activation and in CV/GF show a negative correlation. (*K*) Scatterplot of log_2_ fold change levels for genes significant (<.005 *P* adjusted) in 24-hour fast/ad libitum versus PPARA activated genes.^48^ (*L*) Scatterplot of log_2_ fold change levels for genes significant (<.005 *P* adjusted) in 24-hour fast/ad libitum versus GF+HFM/GF shows many of the same PPARA targets are activated by fasting and HFM.^48^

We conducted overlap analysis of significantly differential H3K27ac sites in each comparison and discovered an interesting property at the +HFM-up separating sites (Figure 4*I*). Rather than preferentially overlap with other +HFM sites, GF+HFM/GF-up sites overlapped with CV+HFM/GF+HFM-down sites, and CV+HFM/CV-up sites overlapped with CV+HFM/GF+HFM-up sites (Figure 6*A*). In fact, almost half (673/1375, 48.9%) of the +HFM-up H3K27ac sites were also microbially responsive (Figure 6*B*). We partitioned these H3K27ac sites into 2 groups: +HFM-up and +CV-up (red) and +HFM-up and +CV-down (blue) (Figure 6*A* and *B*). On the basis of our initial identification of proliferation and ISC genes neighboring CV+HFM/CV-up sites (Figure 4*H–K*), we asked whether these red sites were associated with the crypt-villus and proliferation-differentiation axis. Using a published dataset,^45^ we found that the +HFM-up and +CV-up H3K27ac sites were rarely accessible solely in enterocytes and instead were frequently enriched only in ISCs, whereas the +HFM-up and +CV-down group frequently contained H3K27ac sites that were enriched in enterocytes (Figure 6*C*). +HFM-up and +CV-up (red) sites were enriched in processes including regulation of the immune system (*Myc*, *Mmp14*, *Pglyrp1*, and *Stat3*), regulation of epithelial cell differentiation (*Arntl*, *Mycn*, *Pax6*, and *Dmbt1*), and Notch signaling (*Hes7*, *Notch1*, *Notch2*, and *Sox9*) (Figure 6*D* and *E*, Supplementary Table 3). We saw limited enrichment for terms involved in catabolism, with the notable exceptions of the glycolysis enzyme *Hk2* and limited FAO components *Cpt1a*, *Cpt2*, *Ppard*, and *Prdm16*. Several metabolic genes leading to lipid synthesis *Fads1*, *Fads2*, *Fasn*, *Dgat2*, and *Acacb* were only present in this +HFM-up +CV-up group, with *Acacb* thought to inhibit FAO in favor of lipid anabolism (Figure 6*E*, Supplementary Tables 3 and 4).^46^^,^^47^ Collectively, these +HFM-up and +CV-up (red) sites and their associated genes are indicative of a relative surplus of energy, lipogenesis, and proliferation in CV+HFM (Figure 6*E*).

We then looked at different combinations of H3K27ac sites that responded to both microbial and nutritional signals and the expression of their neighboring genes in a previously published dataset comparing small intestinal enterocytes and ISCs (Figure 6*F*).^45^ The +HFM-up and +CV-up (red) sites were linked to genes expressed preferentially in ISCs relative to enterocytes (Figure 6*F*). Importantly, including +HFM-up and +CV-down (blue), no other groups of sites that responded to both +HFM and +CV signals were associated with ISC and enterocyte progenitor genes (Figure 6*F*).

**Figure 7:**
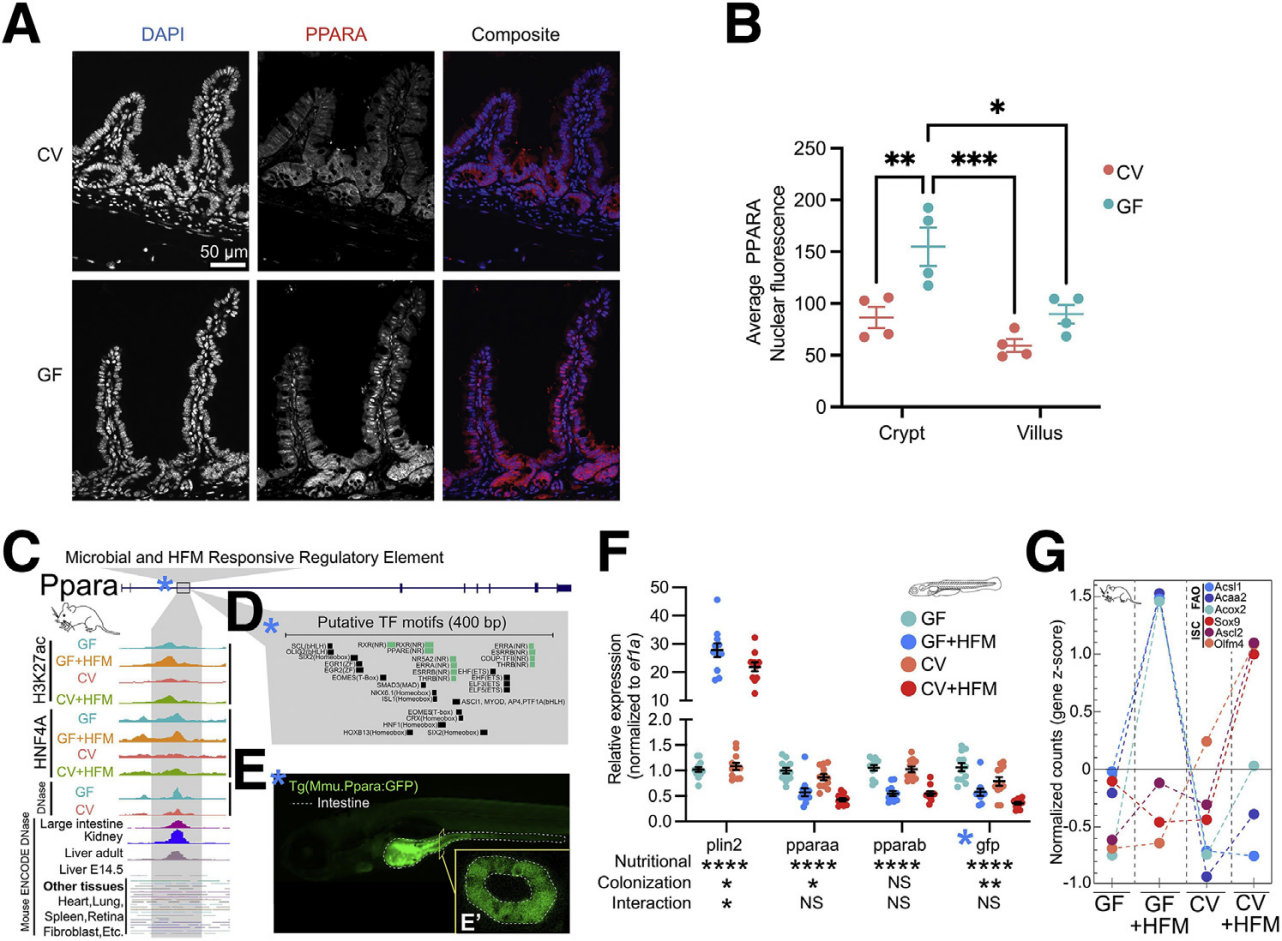
**Characterization of the putative PPARA regulatory region that responds to microbes and HFM.** (*A*) PPARA immunofluorescence of small intestinal villi (*red*) in GF and CV mice fed ad libitum. (*B*) Quantification of IEC PPARA nuclear fluorescence for the crypt and villus identifies higher nuclear fluorescence in GF mice. ∗*P* value ≤.05, ∗∗*P* value ≤.01, and ∗∗∗*P* value ≤.001; two-way ANOVA; n = 4 per condition. (*C*) Exploded view of H3K27ac region at mouse *Ppara* locus from Figure 6*G* that is +HFM-up and +CV-down showing signal across conditions for H3K27ac, HNF4A, and accessible chromatin for jejunum and numerous other tissues. (*D*) Putative TF motifs at the mouse *Ppara* regulatory region include multiple nuclear receptor sites, including PPARE. (*E*) Transgenic *Tg*(*Mmu.Ppara:GFP*) zebrafish at 6 days post-fertilization (dpf) with the mouse *Ppara* regulatory region upstream of a mouse *cFos* minimal promoter driving GFP shows expression largely limited to the anterior intestine. E' boxed inset shows a confocal cross section confirming the signal is specific to IECs. (*F*) Quantitative real-time polymerase chain reaction of a 4 condition experiment using whole 6 dpf *Tg*(*Mmu.Ppara:GFP*) zebrafish shows similar responses to colonization and HFM for *pparaa* and *gfp*. Significance calls for colonization, nutritional, and interaction based on 2-factor ANOVA: ∗*P* value ≤.05, ∗∗*P* value ≤.01, and ∗∗∗∗*P* value ≤.0001. Ten to 20 larvae per replicates; 11-12 replicates per condition. (*G*) FAO and ISC associated genes showing particular expression in IECs from GF+HFM and CV+HFM mice, respectively (Supplementary Table 2).

In contrast, genes neighboring H3K27ac sites that were +HFM-up and +CV-down (blue) (Figure 6*B*) were enriched for lipid metabolism GO terms such as cellular lipid catabolic process and FAO (Figure 6*D*, Supplementary Table 3). The GO terms included genes *Angptl4*, *Acaa2*, and *Acsl5* and the gluconeogenic regulator *Pck1*, which generally had similar patterns at the level of RNA-seq and H3K27ac (Figure 6*G*). In addition, a regulatory region at the FAO regulator, *Ppara*, was in the +HFM-up and +CV-down group (Figure 6*G*). FAO genes and other genes associated with energy production are transcriptionally activated by the lipid-liganded PPARA to facilitate the production of adenosine triphosphate from lipids.^49^ Only +HFM-up and +CV-down H3K27ac sites were substantially linked to published jejunal PPARA target genes (Figure 6*H*).^50^ Consistent with this finding, comparing expression levels after PPARA activation versus HFM response or microbial response revealed a positive and negative correlation, respectively (Figure 6*I* and *J*). In addition, many of the same genes induced by HFM were also induced by fasting (Figure 6*K* and *L*).^48^ We speculated that PPARA may be elevated in GF and that this may help explain why GF+HFM results in relatively high activation of PPARA genes relative to CV+HFM and how PPARA may integrate nutritional and microbial signals. In accord, we identified increased intestinal PPARA protein levels in GF by immunofluorescence (Figure 7*A* and *B*).

### Mouse Ppara Enhancer Shows Evidence of Conserved Intestinal Expression and Microbial and HFM Responsiveness in Transgenic Zebrafish

We hypothesized that the *Ppara* H3K27ac enhancer we identified may be responsible for regulating *Ppara* in IECs and capable of regulating *Ppara* expression in response to microbes and HFM (Figure 6*G*). The *Ppara* enhancer is bound by HNF4A, displays accessibility largely restricted to adult digestive tissues, and contains a PPARE and other nuclear receptor binding sites (Figure 7*C* and *D*). Stable transgenic zebrafish expressing green fluorescent protein (GFP) under control of the *Ppara* mouse enhancer drove expression largely in IECs (Figure 7*E*). We found that as in mouse, both zebrafish *pparaa* and GFP were down-regulated after colonization (Figure 7*F*). However, the GFP reporter or zebrafish *pparaa* was instead concordantly reduced by HFM after 6 hours in zebrafish, suggesting potentially conserved regulation but lacking induction by HFM at this time point.^18^^,^^51^ Collectively, this suggests that HFM preferentially influences H3K27ac sites around genes involved in ISCs and proliferation with microbes and PPARA signaling and FAO without microbes in mouse. This effect can also be seen at key FAO and ISC markers at the level of RNA (Figure 7*G*).

**Figure 8:**
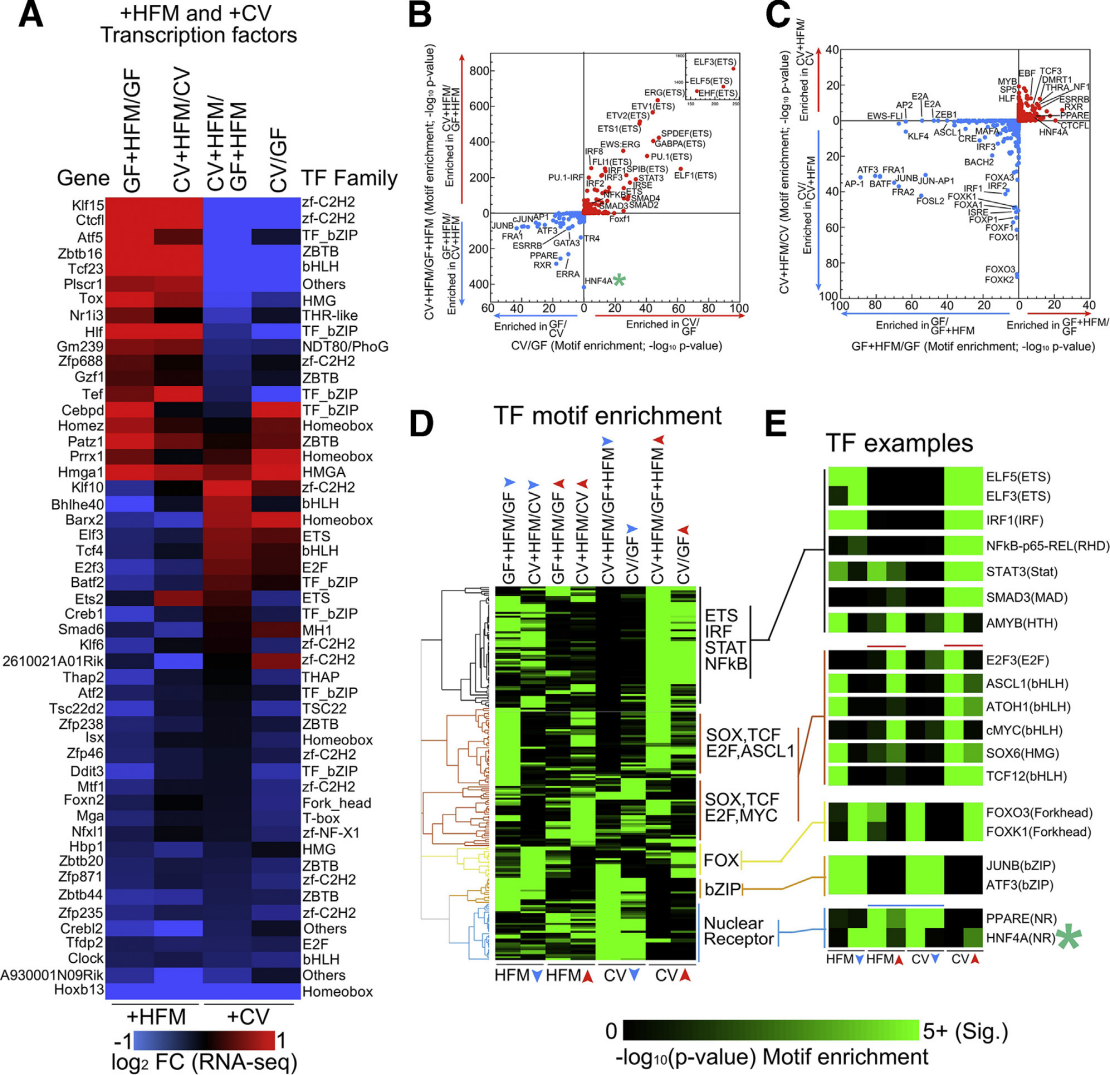
**Enrichment of TF motifs implies HNF4A distinguishes sites that are differential between CV+HFM and GF+HFM.** (*A*) Heatmap of TFs that are significantly different in at least one +HFM and one +CV comparison by RNA-seq. (*B*) Motif enrichment at DNase sites linked to +CV H3k27ac significance groups (*P* adjusted <.05) comparing CV/GF and CV+HFM/GF+HFM sites. For each significance +CV group the reciprocal direction is used as the background (e.g., CV/GF-up input versus CV/GF-down background). The –log_10_*P* values are plotted for motif enrichment. Both directions are plotted on the same axis with each analysis separated by *colored arrows*. HNF4A motif (*green asterisk*) was not differentially enriched between CV/GF directional H3K27ac sites but was substantially enriched in CV+HFM/GF+HFM-down sites relative to CV+HFM/GF+HFM-up sites. (*C*) Same as (*B*) for GF+HFM/GF versus CV+HFM/CV. (*D*) Clustering of enrichment motif score (–log10 *P* value) for TF motifs that are present in multiple comparisons for 8 directional significance groups. Data are shared with (*B*) and (*C*). (*E*) Example TF motif enrichment patterns including those coincident with red (+HFM-up and +CV-up) and blue (+HFM-up and +CV-down) H3K27ac sites.

Other regulatory region and gene groups that respond differentially to microbes and HFM are likely important. A subset of genes were linked to H3K27ac sites that are differentially enriched in +HFM-down as well as either +CV-down or +CV-up comparisons with similar RNA-seq expression patterns. For example, the circadian-regulated lipid transporter CD36^27^is thought to be a PPARA-activated gene; however, we found both H3K27ac and RNA-seq levels at *Cd36* were reduced by colonization and HFM in our dataset (Supplementary Tables 2 and 4).^26^ These differentially expressed genes may be regulated by TFs, of which many are themselves responsive to both microbes and HFM at the transcript level (Figure 8*A*).

### HNF4A Motif Is Differentially Enriched in Microbially Suppressed H3k27ac Sites Only in the Presence of HFM

We next used TF motif enrichment within differential H3K27ac sites to find potential transcriptional regulators in the 4 conditions (Figure 8*B–E*). Common motifs within +CV-up sites include established microbially responsive transcription factors such as nuclear factor kappa B, IRF, and STAT (Figure 8*B*). We previously showed HNF4A motifs are not significantly differentially enriched between CV/GF groups (Figure 8*B*).^22^ Surprisingly, in the presence of HFM, the HNF4A motif was enriched at sites that have reduced H3K27ac with a microbiota (CV+HFM/GF+HFM-down). We clustered motifs for each comparison to identify patterns of their enrichment in +CV and +HFM conditions (Figure 8*D* and *E*). +HFM-up and +CV-up comparisons included several motifs associated with proliferation including E2F3, ASCL1, and cMYC. Both PPAR and HNF4A showed motif enrichment in +HFM-up and +CV-down comparisons, suggesting they may integrate microbial and HFM signals associated with the blue H3K27ac sites group (Figure 8*B–E*).

### The Association of Hnf4a With the Response to Microbes Is Due to Its Impact on Enterocyte Differentiation and the Crypt-Villus Axis

**Figure 9:**
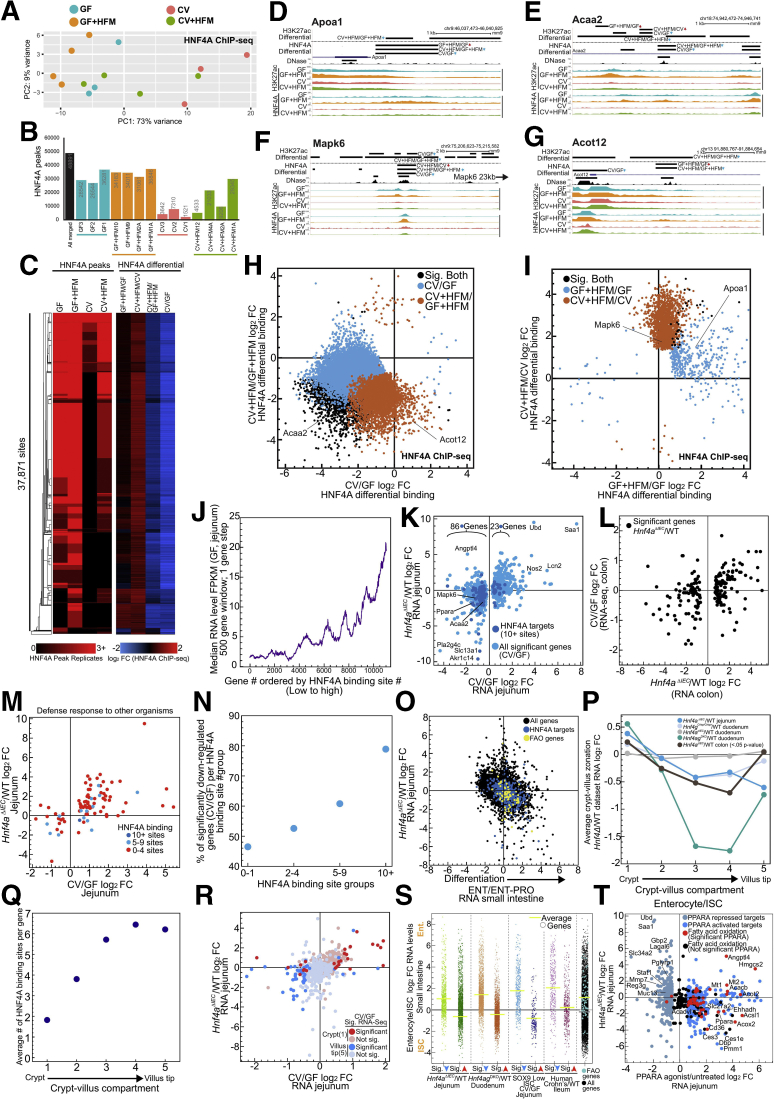
**HNF4A’s role in promoting IEC differentiation explains correlation between *Hnf4a* loss and microbially responsive genes.** (*A*) DESeq2 PCA of HNF4A ChIP-seq normalized counts shows the CV condition deviates from all other conditions. Replicates represent individual mice: CV = 3, CV+HFM = 4, GF = 3, and GF+HFM = 4. Adonis permutational multivariate ANOVA of HNF4A distance matrix; microbes: *P* = .004, R2 = 0 .158; meal: *P* = .136, R2 = 0.094. (*B*) HNF4A peak numbers across GF, GF+HFM, CV, and CV+HFM as well as merged across all replicates and conditions (false discovery rate <0.05). (*C*) Clustered heatmap of HNF4A binding sites with peaks called in at least 2 replicates compared with log_2_ fold change for each of the 4 comparisons. (*D–G*) UCSC screenshot of HNF4A binding sites that are significantly differential, corresponding to (*H*) and (*I)* at the *Apoa1* (*D*)**,***Acaa2* (*E*), *Mapk6* (*F*), and *Acot12* (*G*) loci. (*H*) Scatterplot of significantly different log_2_ fold change for CV/GF and CV+HFM/GF+HFM HNF4A occupancy (false discovery rate <0.05). (*I*) Same as (*H*) for GF+HFM/GF and CV+HFM/CV HNF4A occupancy. (*J*) Genes ordered by the number of neighboring HNF4A binding sites show a positive correlation with gene expression level. (*K*) Scatterplot comparison between significantly differential microbial responsive genes and *Hnf4a*^ΔIEC/^WT in the jejunum.^54^ Genes with more than 10 neighboring HNF4A sites are indicated (*dark blue*). (*L*) Scatterplot comparing genes that have significantly differential expression in *Hnf4a*^ΔIEC^/WT colon^56^ versus CV/GF colon.^21^ (*M*) Scatterplot of significant CV/GF log_2_ fold change RNA-seq levels versus *Hnf4a*^ΔIEC^/WT RNA levels for genes in the Defense Response GO term are not frequently linked to numerous HNF4A binding sites. (*N*) Percentage of significant CV/GF genes per HNF4A binding site group that are significantly down-regulated for different binding site group bins. (O) Scatterplot comparing small intestine RNA-seq log_2_ fold change for enterocyte/enterocyte-progenitor versus *Hnf4a*^ΔIEC^/WT. 10+ HNF4A binding sites/targets (*blue*) contribute directly and indirectly to FAO genes (*yellow*) activation preferentially in enterocytes.^52^^,^^58^ (P) Comparison of genes that are preferentially expressed in 5 compartments along the crypt-villus axis for various *Hnf4* deletion mutants in the intestine.^30^^,^^54^^,^^56^^,^^57^ (Q) Average number of HNF4A binding sites per gene based on crypt-villus compartment groups.^57^ (*R*) Scatterplot comparing jejunal microbial response (CV/GF) to *Hnf4a*^ΔIEC^/WT in jejunum colored for genes preferentially expressed in the crypt (*red*) and villus tip (*blue*).^54^^,^^57^ (*S*) RNA-seq log_2_ fold change for small intestinal enterocyte/ISC RNA levels for groups of significantly differential down (*blue arrow*) and up (*red arrow*) genes from numerous published datasets showed a common impact of *Hnf4a*^ΔIEC^/WT,^54^*Hnf4ag*^*DKO*^/WT,^30^ CV/GF in sorted ISCs,^58^ and human ileal Crohn’s^59^ on the crypt-villus/proliferation-differentiation axis.^45^ FAO genes are also more highly expressed in enterocytes versus ISCs (Supplementary Table 7). *Yellow bars* refer to the average enterocyte/ISC log_2_ fold change RNA levels for each group. *Gray bar* represents the average for all genes except FAO genes. (*T*) Scatterplot comparing PPARA-activated genes versus *Hnf4a*^ΔIEC^/WT RNA levels in mouse jejunum identifies that PPARA targets and FAO genes are commonly reduced in *Hnf4a*^ΔIEC^.

To understand how HNF4A binding contributes to transcriptional integration of microbial and nutritional signals, we performed HNF4A ChIP-seq in the 4 conditions. HNF4A occupancy in the intestine is known to be reduced at binding sites in the CV condition.^22^ PCA showed that unlike RNA-seq and H3K27ac, HNF4A does not distinguish the 4 conditions along PC1 and PC2, with CV datasets segregating along PC1 (Figure 9*A*). Consistent with this, we saw the number of HNF4A binding sites increase in the CV+HFM condition relative to CV (Figure 9*B*, Supplementary Table 6). Differential HNF4A binding analysis identified distinct changes, with +HFM generally increasing HNF4A occupancy and +CV generally decreasing HNF4A occupancy (Figure 9*C–I*). Although provocative, this behavior appeared to be consistent at most sites genome-wide, limiting our ability to interpret meaningful biological differences in HNF4A occupancy levels from individual sites (Figure 9*C*, *H*, and *I*). We instead used the relatively stable location of HNF4A binding sites across conditions. HNF4A binding sites are numerous in the intestine, with typically 15,000–30,000+ binding sites in IECs.^22^^,^^32^^,^^52^^,^^53^ However, transcription level changes in *Hnf4a*^ΔIEC^ mice have been shown to be correlated with not just binary presence or absence but the cumulative number of neighboring HNF4A binding sites for a particular gene.^53^, ^54^, ^55^ We identify a similar correlation with gene expression levels and neighboring HNF4A binding site number (Figure 9*J*).

Using a previously published dataset of mice lacking *Hnf4a* in jejunal IECs (*Hnf4a*^ΔIEC^),^54^ we found that HNF4A-activated genes are also more highly expressed in the GF condition relative to CV (Figure 9*K*). That suggests the relationship found in zebrafish between the HNF4A regulon and microbially responsive genes is conserved in mouse jejunum, with a similar relationship also seen in mouse colon (Figure 9*L*).^22^ We also saw that genes up-regulated in *Hnf4a*^ΔIEC^ mice are correlated with those up-regulated after colonization (Figure 9*K*), with defense response related genes such as *Saa1* being consistently up-regulated by *Hnf4a* loss and presence of microbes across species (Figure 9*K* and *M*).^22^

We found that direct target genes with abundant neighboring HNF4A binding sites (10+ binding sites) are more likely to lose expression in both *Hnf4a*^ΔIEC^ and after colonization with microbes (Figure 9*K*). Conversely, by this metric, genes induced by microbial colonization and in *Hnf4a*^ΔIEC^ are less frequently direct HNF4A targets, suggesting inflammatory induction after HNF4A loss is not due to derepression of HNF4A targets (Figure 9*K*, *M*, and *N*).^22^^,^^56^ Importantly, even at the remaining loci with little or no evidence of being a direct HNF4A target, a correlation between *Hnf4a*^ΔIEC^ and CV/GF expression levels remained for both up- and down-regulated genes (Figure 9*K*). This implicates direct, indirect, or non-canonical HNF4A functions as contributors to the correlation between HNF4A-regulated and microbially regulated genes in the jejunum. HNF4A function in response to microbiota may also be distributed across multiple regulatory regions for a particular gene.^60^

### Microbial and Nutritional Stimuli Influence the Crypt-Villus Axis Leading to Differential Utilization of HNF4A and Differentiation Programs

We investigated whether more global changes in cell identity or cellular programs explain the relationship between HNF4A and microbial colonization. HNF4A and HNF4G initially were identified as positive regulators of enterocyte differentiation with reduced HNF4A activity in proliferating cells.^52^^,^^61^^,^^62^ HNF4A and HNF4G are believed to work together to promote brush border genes and in regulating FAO in ISCs.^30^^,^^60^ Using previously published datasets, we find genes that are preferentially expressed in terminally differentiated enterocytes relative to enterocyte progenitors are negatively correlated with expression in *Hnf4a*^ΔIEC^ mouse jejunum (Figure 9*O*).^63^ We also find loss of *Hnf4a* resulted in increased expression of crypt genes and a decrease of genes expressed in the differentiating villus in the jejunum, colon, or the simultaneous loss of *Hnf4a* and *Hnf4g* in duodenum (Figure 9*P*).^55^^,^^56^^,^^64^^,^^57^ HNF4A binding site number per gene also showed enrichment in villus-expressed genes with limited HNF4A binding at genes preferentially expressed in the crypt (Figure 9*Q*).^57^ To simultaneously interrogate the impact of HNF4A and microbes on the crypt-villus axis, we overlaid the preferential position of a gene’s expression in 5 zones along the crypt-villus axis relative to microbial response and *Hnf4a* dependence (Figure 9*R*).^57^ Genes that were both microbially suppressed and lose expression in *Hnf4a*^ΔIEC^ were preferentially expressed in the differentiated villus. In contrast, microbially activated genes and those with increased expression in *Hnf4a*^ΔIEC^ coincidentally were preferentially expressed in the crypt, directly implicating HNF4A and the response to microbes in regulating differences in the crypt-villus axis and enterocyte differentiation (Figure 9*R*).

In support of the hypothesis that microbial responses impact the underlying differentiation status of IECs, we queried a published RNA-seq dataset of sorted jejunal ISCs in separate GF and CV conditions. We not only observed the previously identified increased expression of proliferation and ISC genes after microbial exposure but also significantly reduced expression of genes preferentially expressed in enterocytes versus ISCs (Figure 9*S*).^58^ Therefore, even when analysis is constrained solely to a population of ISCs, the ISC-enterocyte differentiation axis can be influenced by microbes and is unlikely to be driven solely by differences in IEC subtype composition between conditions in our experiments. This differential expression of genes along the crypt-villus axis may also explain the relationship between inflammatory phenotypes and HNF4A observed in human Crohn’s disease (Figure 9*S*).^22^^,^^59^

We speculated that HNF4A may promote preferential expression of FAO genes in differentiated enterocytes in the GF and GF+HFM conditions relative to CV+HFM. We found that FAO genes are preferentially expressed in enterocytes versus ISCs and enterocyte versus enterocyte progenitors in normal chow-fed conditions (Figure 9*O* and *S*, Supplementary Table 7). Similarly, loss of *Hnf4a* in jejunum results in loss of expression of FAO genes, PPARA targets generally, and *Ppara* itself (Figure 9*T*).^22^^,^^50^^,^^64^ As a result, some of the capacity for FAO in the jejunum relies on HNF4A potentiating an enterocyte differentiation program upstream of *Ppara* and FAO targets.^30^

### Condition Specific H3k27ac Changes Are Restricted to Enterocyte- and ISC-Specific Regulatory Regions and Impacted by HNF4A Binding

**Figure 10:**
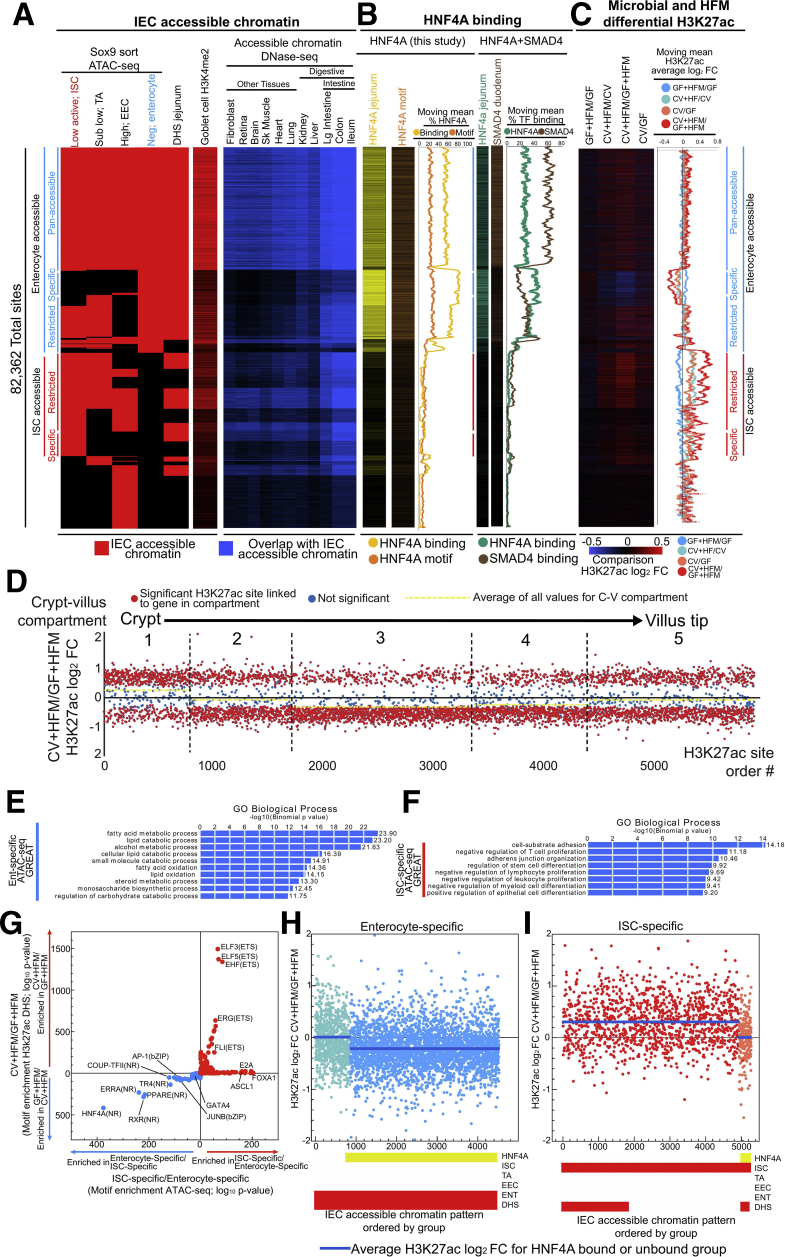
**HNF4A-dominated enterocyte and HNF4A-absent ISC regulatory regions behave distinctly in response to microbes and HFM.** (*A*) Organized heatmap of merged accessible chromatin peak calls for sites that were significantly enriched in at least 1 of the 4 sorted IEC cell types (*red*).^67^ Jejunal DHS sites^22^ and H3K4me2 sites from goblet cells^63^ are also included. Groups of sites that are accessible in enterocytes and ISCs are further broken down into whether they are accessible in other (restricted) or only their IEC type (specific). A subset of sites are accessible across IEC subtypes (pan-accessible). Corresponding accessible chromatin data overlap with other mouse tissues (*blue*) identifies patterns of enrichment.^21^^,^^68^ Groups of sites that correspond to enterocyte sites are marked with a *straight blue line*, and ISC but not enterocytes sites are marked with *straight red lines*. (*B*) Heatmap for HNF4A binding (*yellow*) and computationally detected HNF4A motif (*orange*) for sites ordered as in (*A*). These data are summarized with descending moving means (500 site window, 1 site step). A similar pattern is seen using a previously published jejunum HNF4A ChIP-seq dataset (*teal*).^54^ Duodenum SMAD4 ChIP-seq dataset (*brown*).^55^ (*C*) Heatmap for H3K27ac differential binding for sites ordered as in (*A*). These data are summarized with descending moving means (500 site window, 1 site step). (*D*) The log_2_FC for H3K27ac sites that are significantly different in at least one comparison for the CV+HFM/GF+HFM comparison grouped by their linked neighboring gene into 1 of 5 compartments based on preferential expression along the crypt-villus axis.^57^ Within the compartments, H3K27ac sites are ordered by random. H3K27ac sites that are significant by the comparison on the Y-axis are *red dots*. All nonsignificant sites are *blue*. *Yellow dashed lines* are average for all sites within that compartment. (*E*) GREAT GO term enrichment for enterocyte-specific sites. (*F*) GREAT GO term enrichment for ISC-specific sites. (*G*) Motif enrichment at ATAC-seq sites linked to ISC-specific and Ent-specific groups versus CV+HFM and GF+HFM groups. For each group the reciprocal direction is used as the background (i.e., ISC-specific input versus Ent-specific background). The –log_10_*P* values are plotted. Both directions are plotted on the same axis with each analysis separated by *colored arrows*. (*H*) Groups of accessible sites (*red bars*) further organized by if they are bound by HNF4A (*yellow bars*) show dependence on HNF4A binding for decreased enrichment in CV+HFM/GF+HFM H3K27ac signal at enterocyte-specific sites and increased (*I*) ISC-specific sites on average.

Because 2 hours after a HFM is likely too soon to induce major changes in IEC cell-type abundance,^65^^,^^66^ the enrichment of HNF4A motifs in GF+HFM (Figure 8*B–E*) and differential utilization of enterocyte and ISC regulatory regions in +HFM-up conditions (Figure 4*J* and *K,* Figure 6*C*) may be due to a global change in the utilization of the enterocyte-differentiation transcriptional program within the larger IEC population (Figure 9*S*). We generated a limited intestinal genomics database using a published dataset of accessible chromatin mapping of ISCs, proliferating transit-amplifying cells, enteroendocrine cells, and enterocytes using a SRY-Box Transcription Factor 9 (SOX9) sorting strategy from adult mouse small intestine to identify simple patterns in IEC accessibility (Figure 10*A*).^22^^,^^67^ To test the impact of the ISC-enterocyte differentiation axis, we grouped enterocyte-accessible regions that were also accessible across all other IEC subtype populations (pan-accessible, includes ISC), sites containing enterocyte accessibility and at least one other IEC subtype (enterocyte-restricted), and enterocyte-specific accessible sites (enterocyte-specific) (Figure 10*A*). We similarly grouped ISC-specific and ISC-restricted sites. Using DNase hypersensitivity site (DHS) accessibility from multiple cell types and our published data from colon and ileum, we identified that pan-accessible sites are frequently accessible constitutively across all cell types (Figure 10*A*).^21^^,^^68^

We overlapped these accessible chromatin sites with HNF4A binding from our data and identified that up to 60% of the pan-accessible sites were also bound by HNF4A (Figure 10*B*). Enterocyte-specific sites rose to a substantial 80% overlap with HNF4A binding (Figure 10*B*). In contrast, sites without enterocyte accessibility, including the large group of ISC-specific and -restricted sites, had limited to no HNF4A binding (∼10%) in our IEC preparation (Figure 10*B*). We confirmed this pattern using a published dataset for HNF4A binding in mouse jejunal IECs (Figure 10*B*).^55^^,^^64^ We computationally detected the HNF4A motif at 30% of the sites with enterocyte accessibility and only 10% of the sites without accessibility in enterocytes. This suggests that sites that are accessible in ISC, but not enterocytes, are not typically conventionally bound by HNF4A in any circumstance. SMAD Family Member 4 (SMAD4) has recently been shown to contribute to enterocyte differentiation with HNF4A.^55^ Although we see substantial coincidence of SMAD4 and HNF4A binding, the SMAD4 binding proportion at enterocyte-specific sites is reduced (∼20%) as compared with pan-accessible sites (60%), suggesting HNF4A contributes more exclusively to enterocyte-specific sites (Figure 10*B*).

Finally, to query the impact of microbial and nutritional signals on the different types of regulatory regions, we compared the relative H3K27ac signal for each site for our 4 comparisons (Figure 10*C*). Surprisingly, intestinal accessibility groups had substantially different H3K27ac responses for different comparisons. CV+HFM/CV and CV/GF show reduction of H3K27ac signal at enterocyte-specific sites. This reduction is even more substantial in CV+HFM/GF+HFM, with the GF+HFM/GF comparison increasing. We identified the opposite effect when looking at ISC-specific sites: substantial H3K27ac signal increases in CV/GF, CV+HFM/CV, and especially CV+HFM/GF+HFM, and reduced signal in GF+HFM/GF (Figure 10*C*). This difference implies a major impact of HFM, in the presence or absence of microbes, is manipulation of the related crypt-villus, ISC-enterocyte, or proliferation-differentiation axis (Figure 10*D*). We found that enterocyte-specific sites are enriched near genes involved in carbohydrate metabolism, FAO, and lipid catabolism including *Abcd3, Acaa2, Acox1, Crot, Ppargc1a, Acsl1*, and *Ppara* (Figure 10*E* and *F*). HNF4A and other nuclear receptor motifs that were enriched in GF+HFM regulatory regions are also enriched in these enterocyte-specific sites (Figure 10*G*). Although this property may be attributable to enterocyte-specific regions behaving as a group or reminiscent of a change in the proportional number of a given cell type, we surprisingly found that only enterocyte-specific sites also bound by HNF4A showed altered H3K27ac changes on average (Figure 10*H* and *I*). HNF4A may therefore have a substantial influence on interacting microbial and nutritional enterocyte-specific regulatory regions.

## Discussion

Physiologic responses by the intestine to microbiota and diet must be understood as dynamic processes. The postprandial response typically takes 8 hours for lipid absorption in mice^69^ and can be influenced by host history, including diet, microbes, and circadian rhythm.^2^^,^^23^^,^^25^^,^^69^ Colonization of GF mice leads to transcriptional responses that remain dynamic for weeks and are dependent on the composition of the microbial community.^26^^,^^70^ The intestinal epithelial layer turns over every 3–5 days, with the potential to alter IEC subtypes and crypt-villus architecture while simultaneously making acute transcriptional changes to existing IECs on the order of minutes.^7^^,^^26^^,^^51^^,^^65^^,^^66^^,^^71^, ^72^, ^73^, ^74^, ^75^, ^76^ We aimed here to understand how the intestine integrates 2 distinct conditions simultaneously to coordinate functional genomic activity, and specifically how microbes modify the response to a HFM. Focusing on a single early stage in the postprandial process, we identified a response with substantial complexity but also with clear contributions from PPARA, proliferation pathways, and HNF4A-driven enterocyte differentiation.

At the level of H3K27ac and RNA-seq, our results indicate that PPARA signaling is preferentially engaged after HFM in the absence of microbes. We speculate that the high level of *Ppara* and FAO gene transcription in GF and GF+HFM may contribute to reduced accumulation and absorption of dietary lipid as compared with CV after HFM and may have additional systemic consequences.^2^^,^^19^^,^^20^ Signaling by PPARA and other nuclear receptors has been associated with gene expression in GF that is suppressed by microbes including in IECs from the colon and ileum.^2^^,^^11^^,^^19^, ^20^, ^21^^,^^25^^,^^26^ Microbes may generally impact PPARA signaling along multiple intestinal regions. However, activation of PPARA reduces lipid droplet levels specifically in jejunum, and jejunal microbes fed a high-fat diet are capable of increasing lipid absorption.^2^^,^^77^^,^^78^ Therefore, both host and microbe regional-specific differences may contribute to lipid metabolism responses. Although we measured aspects of the host response to HFM at one early postprandial time point, additional research could uncover the impact of acute HFM on microbiota and how these dynamics change across the prandial cycle and in the context of chronic high-fat diet consumption.

Our results identify FAO as a key process interactively regulated by microbes and HFM, but molecular mechanisms remain unresolved. We were interested to find that the same PPARA-associated genes that are induced by HFM are also induced by fasting (Figure 6*K* and *L*).^48^ Fasting liberates stored fat to produce PPAR-activating FAs that can also be consumed by FAO to produce adenosine triphosphate.^79^ The egg yolk meal used here is rich in FAs, which could act as PPARA ligands.^36^ GF mice may be metabolically primed to initiate FAO to use a HFM as compared with CV mice.^20^^,^^25^^,^^26^ Intestinal FAO and lipid storage might also be influenced by microbial metabolites such as δ-valerobetaine through the inhibition of carnitine synthesis,^80^^,^^81^ acetate through the AMPK/PGC-1α/PPARα signaling pathway, or by c-Jun/NCoR regulation.^12^^,^^20^^,^^25^ We were also interested to see that pharmacologic activation of PPARA not only led to activation of FAO genes that are typically suppressed by microbiota but also suppressed genes normally induced by microbiota such as *Reg3g* and *Saa1* (Figure 6*I*).^50^ This raises the intriguing possibility that PPARA signaling directly opposes proinflammatory programs in the small intestine.^25^

In contrast to PPARA signaling, ISC and proliferation components were preferentially engaged by HFM in the presence of microbes. Increased IEC proliferation has been associated with microbiota colonization in mouse and zebrafish.^26^^,^^58^^,^^82^, ^83^, ^84^ Chronic high-fat diet is also associated with changes in crypt size and increased intestinal proliferation,^15^^,^^75^^,^^76^^,^^85^ and in *Drosophila*, only in the presence of microbes does a high-fat diet induce proliferation in the intestine.^86^ We found that regulatory regions that have increased H3K27ac signal in response to both microbes and HFM (+HFM-up +CV-up) are associated with ISC regulatory regions and expression (Figure 6*C* and *F*). Interestingly, this ISC association emerged despite all our experiments here being from whole jejunal epithelium preparations that do not differentiate between ISC or enterocyte populations. On the basis of typical cell proliferation rates, we consider it unlikely that substantial changes in cell-type abundance occurred within the 2 hours after HFM.^65^^,^^66^ Our analysis of previously published data identifies that unlike CV, isolated GF jejunal ISCs appear to express genes associated with enterocytes relative to ISCs (Figure 9*S*).^58^ Relationships between metabolism and proliferation and differentiation in the intestine are salient and complicated. Loss of *Cpt1a* expression in IECs leads to reduced FAO and proliferation,^87^^,^^88^ and loss of FAO in *Hnf4ag*^*DKO*^ or by chemical suppression is coincident with reduced ISC markers but increased transit-amplifying proliferation markers.^30^ In contrast**,** activation of FAO with a PPARA agonist results in suppression of the MYC proliferation program in the intestine.^50^ It remains unclear whether these impacts of FAO regulation on ISCs and proliferation are direct or indirect. Indeed, PPAR signaling and FAO components are more highly expressed in enterocytes (Figure 9*Q*, *S*, and *T*). The microbial metabolite lactate has been shown to both inhibit FAO and activate intestinal ISCs, consistent with our findings and underscoring that distinct microbial signals can link altered lipid metabolism and intestinal proliferation.^20^^,^^89^

HNF4A may sit at the intersection of microbial and nutritional signals and to intestinal adaptation generally.^90^ Here we identify a conserved correlation between microbially regulated genes and HNF4A-regulated genes in mouse jejunum (Figure 9*K–M*).^22^ We propose that the correlation is driven by the related function of HNF4A promoting enterocyte differentiation relative to proliferation and microbial colonization promoting proliferation relative to differentiation. In addition, the potential for *Hnf4a* loss to eventually lead to intestinal inflammation may compound similarities to microbial responsive genes.^22^^,^^56^ HNF4G, which also contributes to enterocyte differentiation and binds at the same sites as HNF4A in IECs, is transcriptionally microbially suppressed in the CV+HFM/GF+HFM comparison (Supplementary Table 2).^22^^,^^55^^,^^67^^,^^91^ HNF4A is proposed to function primarily as an activator genome-wide in IECs, although it can also function as a repressor and is expressed in both enterocytes and ISCs.^30^^,^^92^, ^93^, ^94^, ^95^ High-fat diet has also been shown to impact HNF4A in the colon and liver.^32^^,^^96^ It is not clear what alters H3K27ac levels in our experiment, although TF candidates such as HNF4A recruit histone deacetylases and histone acetyltransferases.^97^^,^^98^

In endodermal organs such as liver and intestine, HNF4A is a known regulator of lipid metabolism^29^^,^^30^ through its regulation of apolipoprotein genes,^31^^,^^99^^,^^100^ the lipid transporter *Cd36,*^95^ and *Ppara,*^95^^,^^101^ many of which are linked to absorptive enterocyte differentiation.^60^ Previous studies identified reduced intestinal lipid uptake in *Hnf4a*^ΔIEC^ mice^33^^,^^102^ and reduced FAO and increased lipogenesis in *Hnf4ag*^*DKO*^, although these mice also have reduced enterocyte differentiation.^30^^,^^55^ We also find HNF4A promotion of enterocyte differentiation may expose a reservoir of lipid-liganded NR binding sites at the genes that regulate lipid catabolism and FAO that are suppressed by microbes, especially after a HFM (Figure 10*A*, *B*, and *G*). HNF4A therefore may potentiate FAO genes by activating PPARA in enterocytes and IECs and could serve as a pathway to modulate intestinal and systemic metabolism (Figure 9*T*).

We were intrigued to find that the gene expression patterns that characterize ISC-enterocyte differentiation that are so heavily dependent on *Hnf4a* and *Hnf4g* in mice are also suppressed in the context of human Crohn’s disease (Figure 9*S*).^22^^,^^59^ Genetic variants at human *HNF4A* are associated with both Crohn’s disease and ulcerative colitis,^103^, ^104^, ^105^, ^106^ and HNF4A is predicted to bind many inflammatory bowel disease–linked cis-regulatory regions and to regulate inflammatory bowel disease–linked genes.^59^^,^^107^ The mechanisms by which microbes, diet, and inflammation regulate intestinal HNF4 activity may therefore represent new prognostic or therapeutic targets for the human inflammatory bowel diseases.

## Methods

### Mouse Husbandry

All mice used in this study were in the C57BL/6J strain originally sourced from Jackson Laboratories (Bar Harbor, ME) and maintained in the National Gnotobiotic Rodent Resource Center at the University of North Carolina (UNC) at Chapel Hill. Male mice were reared under GF conditions or reared GF and colonized for 14 days with a conventional microbiota from feces of C57BL/6J SPF mice (CV). Mouse colonization was performed exactly as previously described.^22^ Whereas conventionalization of GF mice stimulates temporally dynamic host responses in the intestine,^26^^,^^70^^,^^108^ we elected to focus on 14 days after colonization for our endpoint because it is sufficient for multiple rounds of epithelial renewal and for the initiation of a robust functional genomic host response.^11^^,^^21^^,^^109^^,^^110^ Production, colonization, maintenance, feeding, and sterility testing of GF mice were performed using the standard procedures of the National Gnotobiotic Rodent Resource Center. Animals were housed on Alpha-dri bedding (Shepherd Specialty Papers, Richland, MI) and fed 3500 Autoclavable Breeder Chow (ProLab, Fort Worth, TX) or Picolab mouse diet 5058 (LabDiet, St Louis, MO) ad libitum. All experiments using mice were performed according to established protocols approved by the Institutional Animal Care and Use Committee at University of North Carolina at Chapel Hill (protocol #12-300.0). All mice in this study were given ad libitum access to food until euthanasia. Because microbiota impacts circadian rhythms,^23^^,^^25^^,^^27^^,^^111^ we were careful to perform mouse endpoints and sample collections at the same time of day.

### BODIPY-Egg Yolk Preparation and Gavage

An egg yolk BODIPY labeled mixture was prepared as described with the following modifications.^112^ A 50% egg yolk mixture was generated using 2 mL of egg yolk (grocery store bought) mixed with 2 mL phosphate-buffered saline. The mixture was sonicated and strained as described to produce liposomes. One hundred eighty-two μL of BODIPY 558/568 C12 (Invitrogen, Waltham, MA; D3835) was air dried and resuspended in 50 μL of 100% ethanol and added to 2.3 mL of the 50% egg yolk liposomes mixture. Two hundred μL of mixture was used for gavage at 8:30 am, and gavaged mice were euthanized for tissue harvest exactly 2 hours later.

### Imaging and Quantification of BODIPY Labeled FAs in Villi

Image analysis was blinded and performed using FIJI. Z-stack confocal images of fixed, flayed open, and whole-mounted duodenal intestinal segments were used for analysis. Z-stacks were taken across the axial plane of upward pointing villi. For each z-stack image, 3 planes were chosen for analysis, one near the top of the villus, one near the middle, and one toward the bottom villus. No 2 planes of each z-stack included the same cells. Villi were selected for analysis on the basis of perfect or near-perfect vertical orientation to avoid analyzing cells from bent or angled villi. From each villus selected for analysis, 3 regions of interest (ROIs) were defined that encapsulated about 5–15 epithelial cells but mostly excluded surrounding tissues or dark spaces. Sections of villi without clearly labeled nuclei were not selected as ROIs because they could represent damaged or poorly imaged parts of the villus. For each villus quantified, 1 additional ROI within the same plane was taken of an empty space to be used as a baseline fluorescence value for normalization. Mean gray value of BODIPY-FA conjugate was measured in each ROI. Mean gray value from nearest control ROI was subtracted from the mean gray value of each villus ROI. The resulting background subtracted mean gray values were averaged for each villus or mouse. Two-sample *t* test was performed using JMP software to test for significant differences at both the per mouse and per villus level. Images with villus BODIPY-FA values closest to the average per-villus BODIPY-FA value for each group were selected as representative images.

### PPARA Immunofluorescence and Quantification

Mouse small intestinal (jejunum and ileum) tissue was dissected and immediately fixed overnight in zinc buffer formalin and then embedded in paraffin. Five-μm-thick paraffin sections were used for immunofluorescence experiments. Sodium citrate buffer (pH 6.0) was used for heat-induced antigen retrieval. Slides were blocked with 10% goat serum in phosphate-buffered saline and then incubated with Rabbit Anti-PPARA (Invitrogen; PA1-822A) diluted 1:100 in antibody dilution buffer (phosphate-buffered saline, 1% bovine serum albumin, and 0.0025% Triton X-100) overnight at 4°C. Slides were washed with TBST (0.1% Tween-20) and incubated with Goat Anti-Rabbit Alexa Fluor 568 (Invitrogen; A-11011) diluted 1:200 in antibody dilution buffer. Slides were washed in TBST and counterstained with DAPI, and coverslips were mounted with ProLong Gold antifade reagent (Invitrogen; P10144). Slides were imaged on a Zeiss Axio Imager Z2 (Oberkochen, Germany) upright microscope with an apotome for optical sectioning in the Duke Light Microscopy Core Facility. Nuclear PPARA fluorescence was quantified in the crypt and villus regions using ImageJ (National Institutes of Health, Bethesda, MA) software. Two-way analysis of variance (Prism, Irvine, CA) was used to analyze data (n = 4).

### Isolation of Intestinal Samples for RNA and ChIP

Jejunal samples were collected and processed exactly as described.^22^ In our hands, these methods yield cell preparations that include most villus epithelial cells, a proportion of epithelial crypts, and a proportion of villus mesenchymal cells (data not shown). Because the vast majority of cells in these preparations are epithelial, we operationally refer to them here as IECs. CV and GF data for RNA-seq, DNase, H3K27ac, and HNF4A ChIP-seq were from Davison et al^22^ (GSE90462), with the exception that 2 additional GF and 2 additional CV RNA-seq replicates were generated for this article. All +HFM replicates were generated for this article.

### RNA Isolation, Library Preparation, and Sequencing

Mouse jejunum IEC samples were subjected to RNA isolation, library preparation, and sequencing as described.^22^ Briefly, before crosslinking, 1/50th of the isolated IECs were suspended in 1 mL TRIzol and stored at –80°C. Thawed IECs in TRIzol were prepared according to the manufacturer’s directions; 200 μL of chloroform was added to the TRIzol, and the sample was vortexed on high for 30 seconds at room temperature. The samples were incubated at room temperature for 2 minutes and centrifuged at 12,000*g* for 15 minutes at 4°C. The top aqueous layer was removed and added to equal volume of isopropanol. The nucleic acids were isolated using a column-based RNA-isolation kit (Ambion, Austin, TX; 12183018A) with an on-column DNase I (RNase-free) treatment (New England Biolabs, Ipswich, MA; M0303L) to remove DNA contamination. RNA was eluted in nuclease-free water, quantified using a Qubit 2.0, and stored at –80°C until submission to the Duke Sequencing and Genomic Technologies Core. RNA-seq libraries were prepared and sequenced by the Duke Sequencing and Genomic Technologies Core on an Illumina HiSeq 2500 (San Diego, CA) for 50 base pair (bp) single end sequencing with 8 samples per lane in the flow cell.

### Chromatin Immunoprecipitation, Library Preparation, and Sequencing

Chromatin immunoprecipitation, ChIP libraries, and sequencing were performed on mouse jejunum IECs as described.^22^ Briefly, frozen and sonicated chromatin from IECs was thawed on ice and diluted in 1 mL of ChIP dilution buffer (1% Triton X-100, 2 mmol/L EDTA, 20 mmol/L Tris-Cl [pH 8.1], and 150 mmol/L NaCl) containing 1× Protease Inhibitor. This mixture was precleared with washed protein G Dynabeads (Thermo Fisher Scientific, Waltham, MA; 10004D) for 3 hours at 4°C with gentle agitation. Beads were removed, and chromatin was transferred to a clean microfuge tube and incubated with a ChIP-grade antibody (4 μg H3K27ac [rabbit anti-H3K27ac, Abcam, Cambridge, UK; ab4729], 8 μg HNF4A [mouse anti-HNF4A, Abcam 41898]) overnight at 4°C with gentle agitation. Antibody-chromatin complexes were pulled down with washed protein G Dynabeads for 4 hours at 4°C with gentle agitation. The antibody-chromatin-bead complexes were washed 5× for 3 minutes with ice cold LiCl wash buffer (100 mmol/L Tris-Cl [pH 7.5], 500 mmol/L LiCl, 1% IGEPAL, 1% sodium deoxycholate) and 1× with ice cold TE buffer at 4°C on a nutator. Washed antibody-chromatin-bead complexes were resuspended in 100 μL of ChIP elution 12 buffer (1% sodium dodecyl sulfate and 0.1 mol/L sodium bicarbonate) and placed in an Eppendorf ThermoMixer C heated to 65°C and programmed to vortex at 2000 RPM for 15 seconds and rest for 2 minutes for a total of 30 minutes. The beads were pelleted, and the supernatant was moved to a new tube. This elution process was repeated once, and corresponding elutions were combined for a total of 200 μL. To reverse crosslinked chromatin, 8 μL of 5 mol/L NaCl was added to each 200 μL ChIP elution and was incubated at 65°C overnight. Immunoprecipitated chromatin was isolated using a QIAquick PCR quick preparation kit (Qiagen, Hilden, Germany; 28104), quantified using a Qubit 2.0 fluorometer, and stored at –80°C until library preparations and amplification. Libraries were always prepared within 3 days of the immunoprecipitation with the NEBNextUltra DNA Library Prep Kit for Illumina (New England Biolabs, E7370S). Prepared libraries were quantified using a Qubit 2.0 fluorometer and submitted to Hudson Alpha Genomic Services Laboratory for 50 bp single end sequencing on an Illumina HiSeq 2500 with 4 samples per lane in the flow cell. GF or CV chromatin for input normalization was generated using the same protocol as above except no antibody was used during the overnight antibody incubation; instead, chromatin was incubated at 4°C with gentle agitation. Bead incubation, reverse-crosslinking, and library preparations for these samples were performed using the same protocol as the ChIPs.

### RNA-Seq Mapping and Differential Expression

Adapter sequences and poor-quality reads were removed from FASTQ files using trime_galore. Trimmed and high-quality sequences were aligned to the mouse genome (mm9) using STAR (version 2.7) using default parameters and length of genomic sequences around annotated junctions equal to 49. Counts per gene for differential expression analysis were generated using HTSeq (version 0.9.1) using default parameters. Differential gene expression analysis was performed using R (version 3.4.1) and DESeq2 (version 1.16.1). Counts from replicates for all 4 conditions were used to generate comparisons for interactions between nutritional status (–/+ HFM) and colonization status (–/+ microbes) using the LRT command from DESeq2. A cutoff of <.05 *P* value and greater than 10 base mean counts was used to identify putative interaction genes. Individual comparisons (e.g., CV+HFM/GF+HFM) were extracted using the Wald test using an adjusted *P* value of <.05 to define differential expression.

### ChIP-Seq Mapping, Peak Calls, and Differential Enrichment

HNF4A, H3K27ac, and input GF+HFM and CV+HFM FASTQ sequences were mapped to mm9 genome using Bowtie2 version 2.3.2 and default parameters. MACS2 version 2.1.1.20160309 callpeak program was used to identify peaks (false discovery rate <0.05) from Bowtie2-generated BAM files for H3K27ac and HNF4A ChIP samples using corresponding GF, GF+HFM, CV, CV+HFM, or input BAM files as a control, using the mouse mappable genome size (option: -g mm) and a bandwidth of 300 bp. Narrowpeaks output was used for HNF4A data. A single bed file of merged HNF4A ChIP peaks and a single bed file of merged H3K27ac ChIP peaks were generated by merging BED files from GF, GF+HFM, CV, and CV+HFM HNF4A replicates and GF, GF+HFM, CV, and CV+HFM replicates H3K27AC, respectively, using bedops v2.4.28 merge function. Blacklisted regions of the mm9 genome were removed from merged bed files using bedtools v2.26.0 subtract function. Merging peaks allowed for identifying changes for the same enriched regions across all 4 conditions and comparisons. To quantify counts for each merged H3K27ac peak for each replicate, sliding windows of 300 bp width and overlapping by 100 bp (200 bp steps) across H3K27ac merged peaks were generated using the R (v3.4.1) package IRanges (v2.8.2) every 200 bp. featureCounts (v1.5.3) was used to generate counts for each H3K27ac window. Each 300 bp H3K27ac window was tested for LRT using counts from replicates for all 4 conditions to generate comparisons for interactions between nutritional status (–/+ HFM) and colonization status (–/+ microbes) using the LRT command from DESeq2. A cutoff of less than .01 *P* value and greater than 15 base mean counts was used to identify putative interaction regions. Individual comparisons (e.g., CV+HFM/GF+HFM) were extracted using DESeq2 using an adjusted *P* value of <.05 to define differential enrichment. Significant windows of differential H3K27ac enrichment were then joined to merged H3K27ac peaks to allow for comparison across conditions. For HNF4A ChIP-seq, featureCounts (v1.5.3) was used to generate counts for each merged peak. DiffBind was used to generate differential peak calls for each comparison (false discovery rate <0.05).

### PCA of Genomics Datasets

PCA plots were generated for RNA-seq, H3K27ac ChIP-seq, and HNF4A ChIP-seq data using normalized counts from DEseq2 and the plotPCA function using default parameters. To determine statistical separation based on condition (microbes, meal), the adonis function from the vegan package (v2.5-7) was used separately with RNA-seq, H3K27ac ChIP-seq, and HNF4A ChIP-seq counts tables.

### DHS Peaks

Merged jejunal IEC DHS peaks from CV and GF replicates were used from Davison et al (GSE90462).^22^

### TF Motif Enrichment

For each H3K27ac ChIP-seq directional-significance-group sequence from the closest jejunal DHS within 1 kb of a H3K27ac site was used for TF motif enrichment analysis. Input was composed of the associated DHS sequences for a single directional H3K27ac significance group. The background was composed of sequences from the reciprocal direction of the input group for the same comparison. TF motif enrichment was generated using the findMotifs command for Homer (v4.10.4) using vertebrate motifs with input and background as described for all 8 H3K27ac directional significance groups.^113^

### Definition of Orthologs

One to one mouse to human orthologs were extracted from Ensembl biomart (Ensembl genes 104) and were used to compare RNA-seq data from ileal Crohn’s disease^59^ with mouse RNA-seq expression levels in enterocytes versus ISCs.^45^

### Identification of Neighboring Genes for H3K27ac, HNF4A, and DHS

To identify nearest neighboring genes (NCBIM37/mm9 Ensembl 91) for different regions of enrichment, ClosestBed was used with the first reported gene used for any ties. To quantify HNF4A sites per gene, each HNF4A site was assigned to a nearest gene, and then the total HNF4A sites per gene was summed. For GREAT analysis, regulatory region coordinates were used as input using default parameters.^44^ GREAT may assign a region to multiple neighboring genes on the basis of definitions that allow gene domains to overlap. These GREAT neighboring gene definitions were used only during reporting about GREAT analysis.

### Venn Diagrams

Venn diagrams were generated using an online Venn diagram maker (http://bioinformatics.psb.ugent.be/webtools/Venn/).

### FAO Genes

FAO genes were defined by extracting genes from FAO associated GO terms. The full list of genes and associations are listed in Supplementary Table 6.

### Kolmogorov–Smirnov

To identify whether a gene’s differential expression level was significantly associated with a neighboring significantly differential H3K27ac site for each comparison, a two-sided Kolmogorov-Smirnov test was performed in GraphPad Prism 9.^22^

### GO Term Metascape

Differential RNA-seq gene lists were used as input for Metascape gene annotation and analysis (https://metascape.org) using default settings. For identification of coincident GO terms, the 8 RNA-seq directional significance gene lists were used as input using the multiple list meta-analysis option and output.^114^

### GREAT GO Terms and Site Distribution

Coordinates for the 8 H3K27ac ChIP-seq directional significance groups or other defined groups were used separately as input for GREAT (http://great.stanford.edu/public/html/index.php) using default settings.^44^ To generate coincident GO terms, exact matches for terms significant in both a +CV comparison and a +HFM comparison were displayed.

### Identification of Motifs in Accessible Chromatin Regions

Genomic sequence from accessible chromatin regions from a merged set of peaks from IEC subtypes^67^ was scored for HNF4A motif presence using the homer2 find command with the HNF4a(NR), DR1/HepG2-HNF4a-ChIP-Seq (GSE25021) position weight matrix. Motif identification in the *Ppara* regulatory region sequence was generated using the homer2 find command and vertebrate known motifs.^113^

### Additional Dataset Use

When possible, available datasets with fully genome-wide results were used as provided in a publication’s supplemental data or on GEO (https://www.ncbi.nlm.nih.gov/geo/). For incomplete microarray experiments, the GEO2R (https://www.ncbi.nlm.nih.gov/geo/geo2r/) analysis function was used with default settings, and unless specified, a <.05 *P* adjusted value was used as a cutoff for significance. For incomplete RNA-seq differential expression data, raw count files were used as input for DESeq2 or EdgeR with default settings. Dataset use is summarized in Supplementary Table 8.

### Direct Infusion MS/MS^ALL^ Lipidomic Analysis

Jejunal IECs were collected and isolated as described,^22^ and IEC pellets were snap frozen and stored at –80°C. IEC samples were transferred to a polytetrafluoroethylene-lined screw-cap test tube containing 1 mL each methanol, dichloromethane, and water. Each mixture was vortexed and centrifuged at 3200 RPM for 5 minutes. The lower (organic) phase was transferred to a new tube using a Pasteur pipette and dried under N_2_. The sample was reconstituted in 600 μL dichloromethane:methanol:isopropanol (2:1:1; v:v:v) containing 8 mmol/L NH_4_F and 20 μL 3:50 diluted SPLASH LipidoMix internal standard. Using established methods,^35^ lipid extracts were infused into a SCIEX TripleTOF 6600+ mass spectrometer (Framingham, MA) using a custom-configured LEAP PAL HTS-xt autosampler with dynamic load and wash (Morrisville, NC). Samples were infused into the mass spectrometer for 3 minutes at a flow rate of 10 μL/min through the electrospray port of a DuoSpray ionization source. The MS/MS^ALL^ data were obtained by acquiring production spectra at each unit mass between 200 and 1200 Da in positive mode. Electrospray voltage was set to 5500 V (and –4500 V in negative ionization mode), curtain gas (Cur) set to 20, Gas 1 and 2 set to 25 and 55, respectively, and temperature set to 300°C. Declustering potential and collision energy were set to 120 V and 40 eV for positive mode ionization. Collision energy spread function was not used because it resulted in incorrect isotope ratios that confounded the isotope correction algorithm in our software. Gas 1 and 2 as well as source gas were zero-grade air, and curtain gas and CAD gas were nitrogen. Processing the lipidomic data was done with in-house software (LipPY) developed at UT Southwestern Medical Center.

### Zebrafish Husbandry

Zebrafish lines were maintained using established protocols approved by the Office of Animal Welfare Assurance at Duke University (protocol #A096-19-04). Conventionally raised zebrafish were reared and maintained as described.^115^ Production, colonization, maintenance, and sterility testing of gnotobiotic zebrafish were performed as described.^116^

### Generation of Tg(Mmu.Ppara:GFP) Transgenic Zebrafish

Molecular biology, injection, and isolation of 2 stable *Tg(Mmu.Ppara:GFP)* zebrafish lines (allele designation *rdu87* and *rdu88*) on an EK zebrafish background was identical to those described previously^42^ using primers listed in Supplementary Table 9. Images and quantitative real-time polymerase chain reaction data from *rdu87* are represented.

### Zebrafish Imaging

Whole-mount 6 dpf zebrafish images were generated on a Leica M205 FA (Wetzlar, Germany) microscope with a Hamamatsu ORCA-Flash4.0 LT (Hamamatsu City, Japan). Two hundred μm axial cross sections of the anterior intestine of 6 dpf crosslinked zebrafish were generated using a Leica VT1000S. Cross-section images were generated using a Leica SP8 (DM6000CS) confocal microscope in the Duke Light Microscopy Core Facility.

### Husbandry, Gnotobiotic, and HFM Zebrafish Treatment

Generation, maintenance, and conventionalization of GF *Tg(Mmu.Ppara:GFP)* zebrafish larvae were conducted as described previously,^117^ with the exception that no exogenous food was administered until 6 dpf. At 6 dpf, half of the larvae of either GF or CV conditions were subjected to a HFM as described,^118^ whereas the other half remained unfed. To feed a HFM, larvae were transferred to sterile 6-well plates (20 larvae/well) and immersed in 5 mL solution of 5% chicken egg yolk liposomes in gnotobiotic zebrafish medium for 6 hours on a rocker at 28.5°C. After feeding, the fed larvae and their unfed counterparts were washed in gnotobiotic zebrafish medium, euthanized, and collected in TRIzol for RNA isolation, respectively (10–20 larvae per replicates; 11–12 replicates per condition).

### Preparation of Zebrafish Tissues and Quantitative Real-Time Polymerase Chain Reaction

mRNA was isolated from each replicate from pooled whole zebrafish larvae as previously described.^119^ RNA concentrations were measured using Thermo Scientific NanoDrop 1000 spectrophotometer and then diluted to match the lowest concentrated sample. The same total mRNA input (300 ng and 800 ng for the 2 independent experiments) was then used for cDNA synthesis using the iScript cDNA synthesis kit (Bio-Rad, Hercules, CA; 1708891). Quantitative real-time polymerase chain reaction was performed in triplicate for each replicate with 25 uL reactions using 2X Sybr Green SuperMix (PerfeCTa, Hi Rox; Quanta Biosciences, Gaithersburg, MD; 95055) with the ABI Quantstudio 3 Real Time PCR system. Data were analyzed with the ΔΔCt method. Gene expression data were normalized using *ef1a* as the housekeeping gene. All statistical tests on quantitative real-time polymerase chain reaction data were performed using Graphpad Prism v.9. Statistically significant effects of diet and microbial colonization on gene expression were determined by performing a 3-factor repeated measure analysis of variance (ANOVA) (gene × nutritional status × colonization), followed by a 2-factor ANOVA for each gene. Post hoc unpaired 2-sided Student *t* tests were applied to each gene exhibiting a significant nutritional status vs colonization interaction.

## Supplementary Data

Note: to access the supplementary materials accompanying this article, visit the online version of *Clinical Gastroenterology and Hepatology* at www.cmghjournal.org.
